# Liquid Metal Gallium Pharmaceuticals

**DOI:** 10.7150/thno.116184

**Published:** 2025-08-11

**Authors:** Dawei Wang, Wei Xu, Yuxin Liu, Luying Chen, Ting He, Hong Tan, Shuting He, Jie Zhu, Caiting Wang, Zhongyang Yu, Chong Li, Weidong Pan, Wei Rao, Jing Liu

**Affiliations:** 1 Key Laboratory of Plant Resource Conservation and Germplasm Innovation in Mountainous Region (Ministry of Education), School of Pharmaceutical Sciences, Guizhou University, Guiyang 550025. Guizhou Province, China.; 2 Guizhou Provincial Key Laboratory of Innovation and Manufacturing for Pharmaceuticals/Guizhou Engineering Laboratory for Synthetic Drugs, School of Pharmaceutical Sciences, Guizhou University, Guiyang 550025. Guizhou Province, China.; 3 Oncology Department, Dongfang Hospital, Beijing University of Chinese Medicine, Beijing, 100078, China.; 4 Graduate School, Beijing University of Chinese Medicine, Beijing, 100029, China.; 5 School of Pharmaceutical Sciences, Southern Medical University, Guangzhou 510515, China.; 6 College of Pharmaceutical Sciences, Southwest University, Chongqing 400716, China.; 7 State Key Laboratory of Cryogenic Science and Technology, Technical Institute of Physics and Chemistry, Chinese Academy of Sciences, Beijing 100190, China.; 8 School of Future Technology, University of Chinese Academy of Sciences, Beijing 100049, China.

**Keywords:** Gallium, Liquid Metal, Pharmaceuticals, Compounds, Complexes, Radioisotopes, Nanomedicines

## Abstract

The room temperature liquid metal gallium, as a multi-targeted pharmacologically active metallic element with long historical investigation, has shown great potential in the field of medicine, especially in antibacterial, anti-inflammatory, anticancer, osteogenesis, radio-pharmacology, molecular imaging and more emerging drug categories. However, diverse structures and physical/chemical compositions, complex interactions with living organisms, and insufficient mechanism interpretation, pose serious challenges for the clinical translation of gallium-based pharmaceuticals. This review systematically identified and described promising gallium-based pharmaceuticals, with emphasis on typical features, fundamental pharmaceutical activities, representative pharmaceutical formulations, and practical implementations, aiming to inspire innovative concepts for drug discovery and further investigation. Meaningfully, new insights into gallium-based liquid metals for screening pharmaceutical formulations are presented, which promise to bring broader strategies for enhancing the bioavailability, targetability, biocompatibility and pharmacological efficacy of active pharmaceutical ingredients (Ga(0)/Ga(III)). Besides, the unresolved challenges and future perspectives of these emerging gallium-based pharmaceuticals were also outlined, aiming to aid for future academic explorations and translational medicine of gallium-based pharmaceuticals. Overall, gallium-based pharmaceuticals with tremendous pharmaceutical formulations and pharmaceutical activities open up a huge scope great potential for future pharmaceutical engineering, which are promising to usher epochal metallodrug development with a series of breakthrough discoveries and pioneering technologies.

## Introduction

In the last decades, the concept of “metallodrugs” has gradually been emphasized, since the success of cisplatin, auranofin and their derivatives 1, 2. The room temperature liquid metal gallium, a rare metallic element, occupies an important role in the electronics industry, which is predominantly used in semiconductors, light-emitting diodes and solar energy applications. However, as seldomly recognized, gallium is also important in medical and pharmaceutical sciences, mainly because of their specific physicochemical properties, favorable biosafety and unique pharmaceutical activities 3-7.

**Figure 1:**
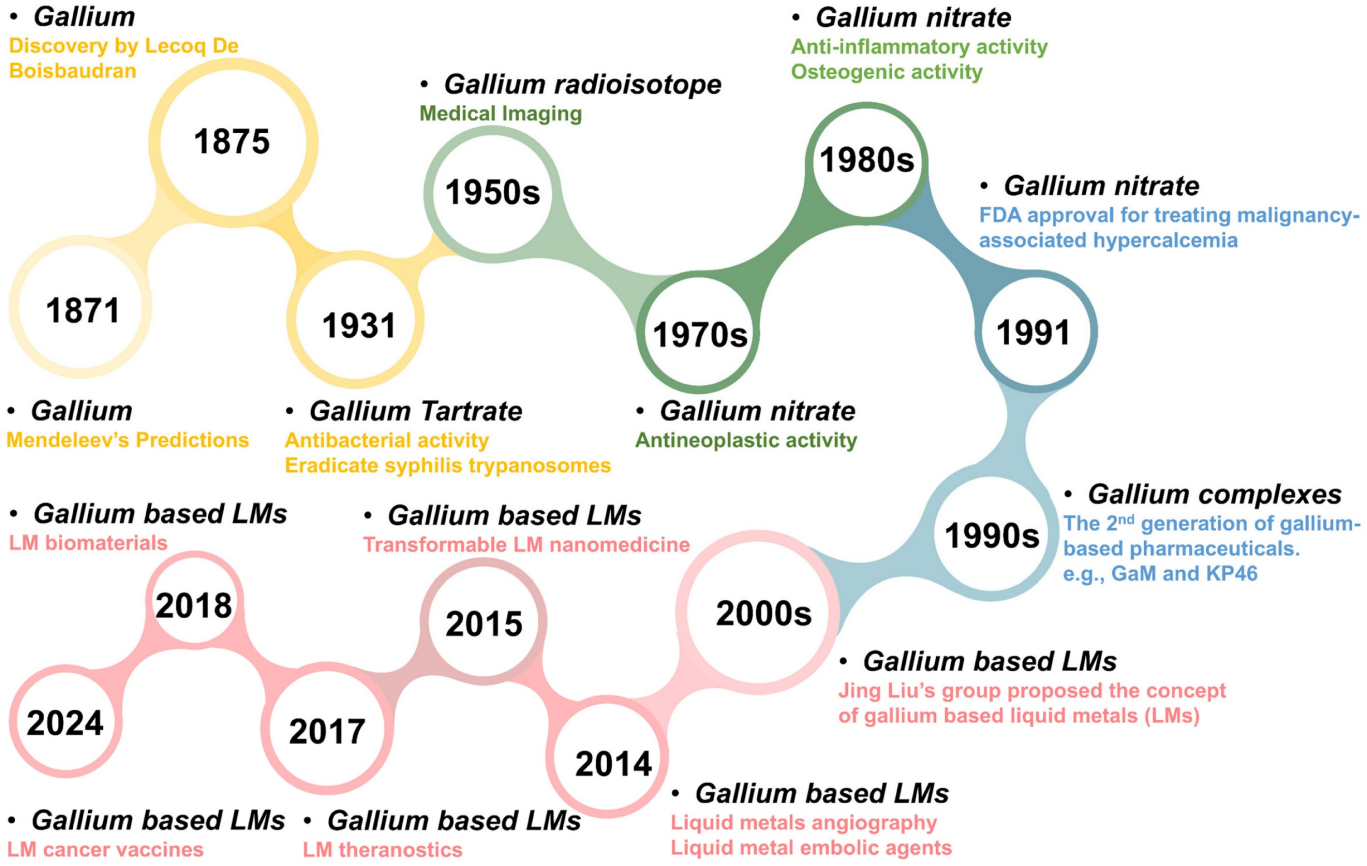
The timeline for the development of gallium-based pharmaceuticals.

In the biomedical field, gallium has been regarded as the second metal after platinum with long historical investigation 8, since gallium and its related derivatives are widely used for malignancy-associated hypercalcemia, radio-pharmacology and molecular imaging 3, 4, 6-10. As early as 1931, Levaditi et al. revealed that gallium tartrate could eradicate syphilis in rabbits and trypanosomes in mice 11. Subsequently, further evaluation of gallium as a potential therapeutic agent appears to have stalled 12. Until the late 1970s, the gallium nitrate entered clinical trials as a National Cancer Institute (NCI)-designated investigational drug (NSC 15200), since its antineoplastic activity was demonstrated in rodents 8. In 1991, the gallium nitrate became the first gallium (III) compound that received Food and Drug Administration (FDA) approval, for the clinical treatment of malignancy-associated hypercalcemia 8, with superior efficacy and safety compared to traditional drugs (e.g., glucocorticoids, pamidronate, calcitonin, and bisphosphonates) 13-15. Since then, the preclinical and clinical progress continues unabated, ranging from simple gallium salts to more structurally complex gallium complexes, with more diverse therapeutic activities being explored against cancer, infection, and inflammation, etc 4, 6, 7, 9, 12, 16, 17. Apart from the therapeutic activities, the diagnostic activities of gallium radioisotopes are also impressive. In the 1950s, gallium's applications in medical imaging were initiated, since researchers discovered that the radioisotope^ 67^Ga would concentrate within implanted tumors in rodents 18. Subsequently, ^68^Ga-labelled pharmaceuticals are emerging for target-specific molecular imaging, which have been approved as positron emission computed tomography (PET) agents in clinical trials 19-22. In addition, more innovative gallium-based pharmaceutical formulations are constantly being developed, such as nanomedicines, nano-sensitizers and nano carriers, which provide strong support for more precise and personalized diagnostic and therapeutic treatments (overview in Figure 1) 23-28.

In recent years, liquid metal gallium and its alloy are receiving ever explosive investigations with many of their physical or chemical properties increasingly disclosed. It is time to revisit, clarify and significantly expend the pharmaceutic roles of gallium. This review is dedicated to systematically identify and describe diverse gallium-based pharmaceuticals, with emphasis on typical features, fundamental pharmaceutical activities, representative pharmaceutical formulations, and practical applications, hoping to inspire innovative concepts for further investigation. The content began with a relatively elaborate description of the basic physicochemical properties, followed by a detailed introduction of the fundamental pharmaceutical activities (e.g., antibacterial, anti-inflammatory, antineoplastic and osteogenic activities). Then, the vision was expanded to representative pharmaceutical formulations, such as compounds, complexes, nanomedicines, sensitizers and bioactive materials, in terms of practical applications for therapeutic agents, diagnostic agents, and drug carriers. Meaningfully, new insights into gallium-based liquid metals (LMs, commonly defined as metals or alloys with melting points below or near room temperature) for innovative pharmaceutical formulations are presented, which are expected to bring broader implications for enhancing the bioavailability, targetability, biocompatibility and pharmacological efficacy of active pharmaceutical ingredients (Ga(0)/Ga(III)). Finally, the unresolved challenges and future outlooks of these emerging gallium-based pharmaceuticals were also summarized. Overall, gallium-based pharmaceuticals with diverse pharmaceutical formulations and pharmaceutical activities hold great potential for biomedicine applications, which are promising to usher epochal metallodrug development with in-depth mechanism exploration and technological innovation.

## Typical Physicochemical Properties

Gallium (Ga, group IIIa metal, atomic number 31, atomic weight 69.72, electron arrangement [Ar]3d^10^4s^2^4p^1^) was the first element in chemical history to be theoretically predicted and then discovered and verified in nature. Since the mid-1940s, gallium and its derivatives (radioisotopes, gallium compounds, gallium complexes, gallium-based LMs, gallium-based bioactive materials, etc.) have attracted a wide range of interest in the biomedical field 10. Meanwhile many specific physicochemical properties and pharmaceutical activities are gradually being explored.

**Gallium:** Gallium is available in the earth's crust in contents of only 0.0015%, and is usually associated with bauxite and lead-zinc ores without forming a separate mineral. In the molten state, gallium appears silver-white in color and turns light blue upon solidification, with a volume expansion of approximately 3.2% 29. Notably, gallium exhibits a pronounced supercooling phenomenon (markedly below its melting temperature), with an even more intensified effect at the micro- and nanoscale levels 17. Compared to the few low melting point metallic elements (below 40 °C), such as mercury (Hg), rubidium (Rb), cesium (Cs), and francium (Fr), elemental gallium is more suitable for biomedical applications due to its non-toxicity (up to 500 mg in healthy humans), non-radioactivity (^69^Ga), and extremely low vapor pressure (~10^-35^ Pa at 30 °C) 5, 30.

**Gallium (III) ion:** The metallic element gallium possesses similar amphoteric chemical properties as aluminum, which makes it soluble in acids and bases accompanied by hydrogen generation 17. While gallium's strong electronegativity and electrochemical potential window may offer remarkable possibilities for potential therapeutic activity, since the *in vivo* electrochemical reactions may disturb ionic equilibrium (reduction potential E^0^: Zn (-0.763V) < Ga (-0.56 V) < Fe (-0.41 V) < Cu (+0.34 V)) and the redox reactions may help to maintain intracellular redox homeostasis 17, 26, 27. In its prevalent cationic state, the gallium ion (Ga³⁺) exhibits analogous chemical behaviors to that of the iron ion (Fe³⁺), notably in their respective octahedral (Ga³⁺: 0.62 Å versus high-spin Fe³⁺: 0.645 Å) and tetrahedral (Ga³⁺: 0.47 Å versus high-spin Fe³⁺: 0.49 Å) ionic radii dimensions. Additionally, their ionization energies (Ga³⁺: 64 eV, as opposed to high-spin Fe³⁺: 54.8 eV) and electron attractions (Ga³⁺: 30.71 eV, akin to high-spin Fe³⁺: 30.65 eV) also display close proximity. However, a significant difference between gallium and iron is that iron is redox-active and can switch between the steady state divalent Fe (II) and trivalent Fe (III). In contrast, the stable valence state of gallium exists only as gallium (III) 12. Consequently, Ga³⁺ possesses the potential to mimic Fe³⁺ in diverse biological frameworks, fostering prospects for therapeutic applications 3, 4, 12.

**Gallium based liquid metals:** Gallium-based LMs (represented by gallium and gallium-indium alloys) can be regarded as amorphous solids in the molten state, and exhibit mobility and metallicity that are fundamentally different from solid metals and other liquids 17, 30. As an emerging class of bioactive materials, gallium-based LMs possess diverse intrinsic characteristics, such as metallic properties (high thermal conductivity, electrical conductivity, and surface tension), amorphous properties (excellent fluidity, flexibility, deformability, and self-healing ability), as well as other favorable properties (biocompatibility, biodegradability, facile functionalization accessibility, solid-liquid phase transition, catalytic properties, and stimuli-responsiveness) 5, 17, 31-37. In recent years, gallium-based LMs have received sustained attention, spawning various groundbreaking frontiers and breakthrough technologies in the biomedical field, including diagnostic agents, nanomedicines, drug delivery carriers, and bioactive materials, etc 5, 6, 32, 34, 38-42.

## Pharmaceutical Activity

Gallium and related derivatives have received much attention due to their excellent biosafety and distinctive pharmacological activities 7. Fundamentally, gallium is a classic multi-target drug that may interfere with iron metabolism/iron homeostasis, dysregulate cellular redox homeostasis and modulate the immune response, thus showing promising antimicrobial, anti-inflammatory, and antineoplastic activity 6, 9, 17, 43. Besides, gallium radioisotopes (^67^Ga, ^68^Ga) and gallium-based LMs are also promising diagnostic agents due to their inherent high density, electromagnetic properties and radioactivity. Moreover, owing to the unique stimulus sensitivity and transformability, gallium-based nanomedicines can not only provide therapeutic activities through heat accumulation, ROS generation, and galvanic replacement, but also serve as innovative responsive drug carriers for spatiotemporally controlled intracellular drug delivery. Hence, gallium and related derivatives have exhibited exceptional pharmaceutical activities in various fields, which deserve intensive research.

### Biosafety

With the increased attention devoted to gallium in the biomedical field, toxicological studies are emerging as an essential issue. Early clinical applications and recent preclinical studies have substantially confirmed that various gallium derivatives, e.g., compounds complexed with nitrate, arsenide, thiosemicarbazone, or maltol, micro/nanoparticles conjugated with drugs, ligands, polysaccharides, or inorganic compounds, as well as macroscopic forms of gallium-based LMs, can be considered with low toxicity in terms of cytotoxicity, hepatotoxicity, hemotoxicity, and histotoxicity 6, 17, 44, 45. In particular, despite the fact that gallium is not an essential element for living organisms, it is generally considered biocompatible and non-toxic at low doses due to its inability to enter erythrocytes (avoiding interference with oxygen transportation), biodegradability in the physiological environment (e.g., Ga^0^ degradation or dissolution *via* redox reactions or oxidative enzymatic reactions, with release of Ga³⁺ ions 37), coupled with efficient excretion through metabolic processes in the form of feces and urine 5, 7, 46. Thus, the virtually non-toxic and biodegradable characteristics of gallium lays the foundation for the safe biomedical applications of related derivatives. However, despite the exponential growth of relevant research, the investigation of the toxicological effects and possible hazards of gallium and related derivatives on living organisms is still in their infancy (for details, see Ref 46).

### Antimicrobial Activity

The emergence of antibiotic resistance and drug-resistant "superbugs" poses a major threat to global public health 47. In response to the growing threat of antimicrobial drug resistance, metallic antimicrobials are flourishing with potent antimicrobial efficacy and favorable safety profile, such as Au, Ag, Cu, Zn, Al, Ga, Sn, and Bi-based metallic antimicrobials. These metallic antimicrobials can be toxic to pathogenic bacteria through various antimicrobial mechanisms, such as disrupting redox metabolic chains, disrupting cell membranes and inducing protein dysfunction 48.

**Figure 2:**
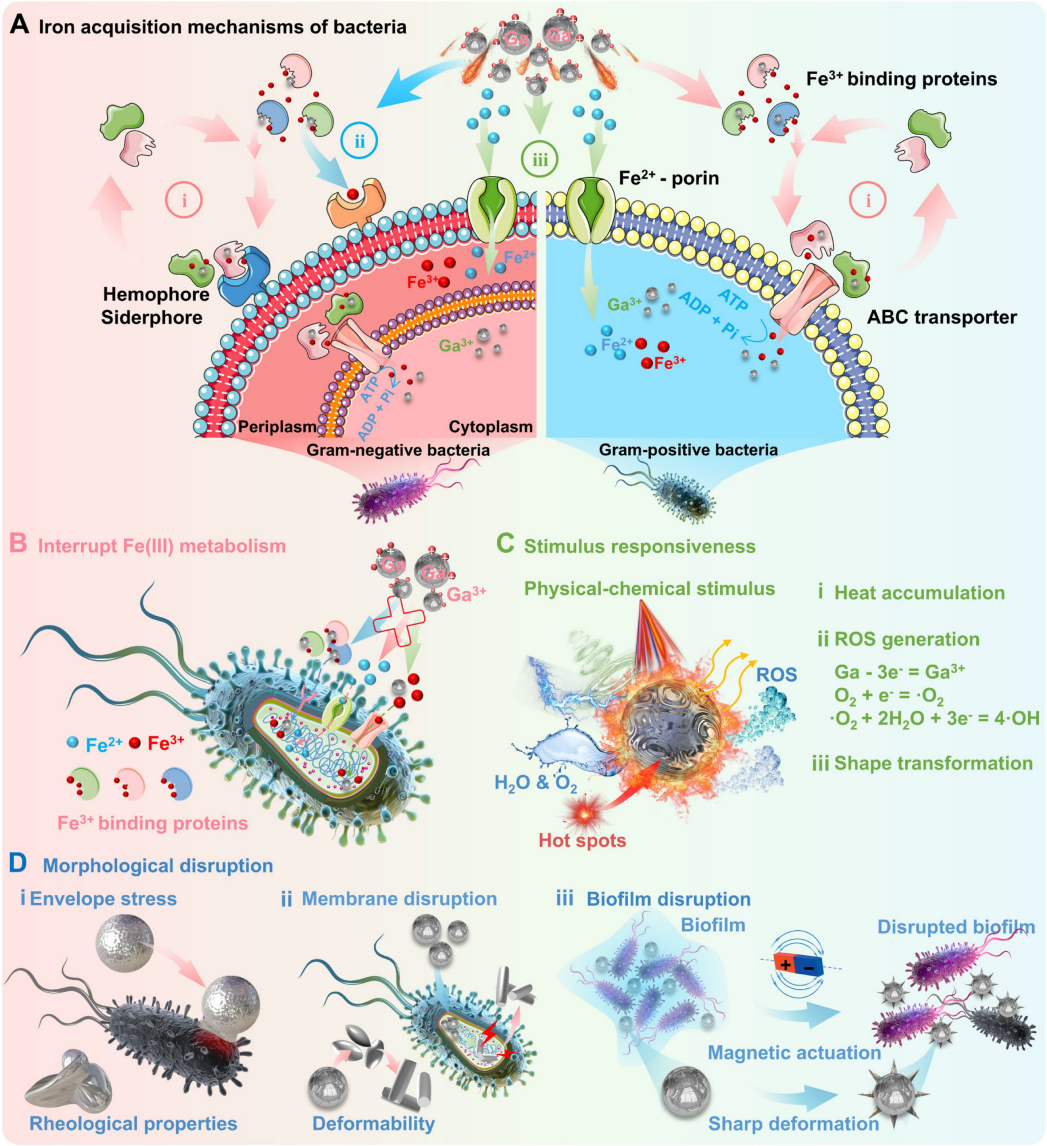
** Representative antimicrobial mechanisms of gallium and its derivatives:** (A) The main iron acquisition mechanisms of bacteria: i) Hemophore/siderophore-dependent uptake systems, ii) Iron uptake systems contain specific surface receptors, iii) Ferrous iron transport systems. Created with Smart.Servier.com. (B) "Trojan horse" strategy, i.e., Ga^3+^ as non-functional Fe^3+^ mimic to effectively interfere with iron metabolism/iron homeostasis, and subsequently triggering downstream effects; (C) Stimulus-response strategy, i.e., inducing ROS generation, heat accumulation, and shape transformation *via* physicochemical stimulus; (D) Mechanical damage strategy, i.e., inducing destructive forces through physical deformation of gallium-based LMs, including envelope stress, membrane disruption, biofilm disruption.

Among them, gallium and related derivatives (in the form of nanomedicines, compounds, complexes, etc.) are the most promising candidates as antimicrobial agents, which may promise impactful and innovative strategies against antimicrobial resistance 48. Iron ions are one of the most essential metal ions required for bacterial survival, involving bacterial cellular respiration, DNA synthesis and reactive oxygen species (ROS) defence 49. Bacteria require access to iron ions to combat iron deficiency, while the major strategies include (Figure 2A): (i) secreting various ferric complexes (e.g., ferric chelating siderophores or hemophores) that bind to Fe(III) for active intracellular transport 50; (ii) acquiring Fe from iron-binding proteins *via* specific transport systems contain specific surface receptors 51; and (iii) direct uptaking Fe(II) *via* the bacterial ferrous iron (Feo) transport systems on the cytoplasmic membrane 52. Thus, a new generation of antimicrobial agents can be developed targeting Fe uptake and metabolism, since iron ions play a crucial role in microbial physiology and pathogenicity. In this regard, gallium and its derivatives as antimicrobial therapeutics can inhibit the growth of common pathogenic bacteria (including *E. coli*, *P. aeruginosa*, *H. influenzae*, *S. aureus* and *A. baumannii*), mainly attributed to the competitive binding between gallium and iron 48. As the most well-known antimicrobial mechanism, Ga^3+^ tend to compete with Fe^3+^ owing to their high degree of similarity 53, in terms of tetrahedral and octahedral ionic radii, ionization potentials, electron affinities, and chemical properties (called "Trojan horse" strategy 7) 6, 49. Unlike iron, gallium cannot toggle between oxidation states once bound to siderophores, since gallium has only one stable oxidation state (Ga^3+^) 48. Therefore, gallium-based antimicrobials are considered to be redox-inert Fe(III) competitive inhibitors, which could effectively interrupt Fe(III) metabolism and lead to pathogenic bacteria death (Figure 2B). In addition to inhibition of iron metabolism 53, the gallium-based antimicrobials can also achieve antimicrobial effects through other mechanisms, e.g., generation of ROS 54, accumulation of heat 55, envelope stress and generation of mechanical disruptions 56 in response to external stimulus (Figure 2C-D).

### Anti-inflammatory Activity

Inflammation is one of the main defensive responses when the immune system against harmful stimuli, but prolonged or chronic inflammatory processes may trigger serious side effects, such as causing tissue damage and destruction, promoting the progression of specific diseases (e.g., cancer, cardiovascular disease, diabetes, etc.), and inducing organ dysfunction or even failure 7. Invasion of pathogens (e.g., bacteria, viruses, fungi, etc.) is one of the main factors triggering the inflammatory response, since the pathogens may cause infection and activate immune cells (e.g. macrophages, neutrophils, etc.) to release a variety of inflammatory mediators (e.g. cytokines, chemokines, etc.) 57. Additionally, pathogens may also stimulate host inflammation utilizing virulence factors and pathogen-associated molecular patterns (PAMPs) 58.

Gallium-based anti-inflammatory drugs are a new class of drugs with unique anti-inflammatory mechanisms, which can exert anti-inflammatory activity through various pathways such as interfering with iron metabolism, generating oxidative stress and modulating the immune response. Gallium compounds, as a source of Ga^3+^ ions, have historically been employed as anti-inflammatory agents in related diseases, such as inflammatory arthritis 59, autoimmune encephalomyelitis 60, and systemic lupus erythematosus 61. It has been demonstrated that Ga^3+^ ions may exert anti-inflammatory effects *via* modulating the production of pro-inflammatory cytokines and nitric oxide (NO) from activated immune cells 59, 62, 63. While, the current rational anti-inflammatory mechanism of Ga^3+^ ions is still a "Trojan horse" strategy, whereby the substitution of Fe^3+^ binding to iron-binding proteins (e.g. transferrin Tf) may impede the native protein functions and further disrupt the iron homeostasis in immune cells 3, 4, 64.

Although this mechanism is effective in suppressing the inflammatory response, it may also disrupt the iron homeostasis of non-targeted cells, thereby affecting normal cell proliferation and differentiation, and inducing adverse downstream effects 4. Recently, nanomedicines constructed from gallium-based LMs have emerged as new pharmaceutical formulations, which not only retain the anti-inflammatory activity of gallium ions, but also significantly improve the biocompatibility and targetability. In contrast to gallium compounds, gallium-based nanomedicines could alleviate inflammation *via* selectively inhibiting NO production without interfering with Fe homeostasis or affecting the accumulation of other pro-inflammatory mediators (e.g. IL-6, TNF-α) 48, 64. Typically, Kalantar-Zadeh, K. et al. found that gallium nanodroplets (GNDs) may inhibit NO production by interfering with the mRNA translation of inducible nitric oxide synthase (iNOS) through the up-regulation of the phosphorylation level of eukaryotic initiation factor-2α (eIF-2α) 64. While the anti-inflammatory activity of GNDs without interfering with normal cellular iron metabolism is closely correlated with the intracellular dissemination route, i.e., gallium-based nanomedicines are uptaken by cells *via* endocytosis rather than relying on the iron transferrin receptor (TfR). In addition, the gallium-based nanomedicines may enable more precise targeted enrichment within inflammatory tissues, leading to more effective alleviation of the local inflammatory response and further reducing potential systemic side effects.

In summary, gallium-based anti-inflammatory drugs with unique anti-inflammatory mechanisms have exhibited great potential for application and will play a more important role in future anti-inflammatory therapy as research progresses.

### Antineoplastic Activity

**Figure 3:**
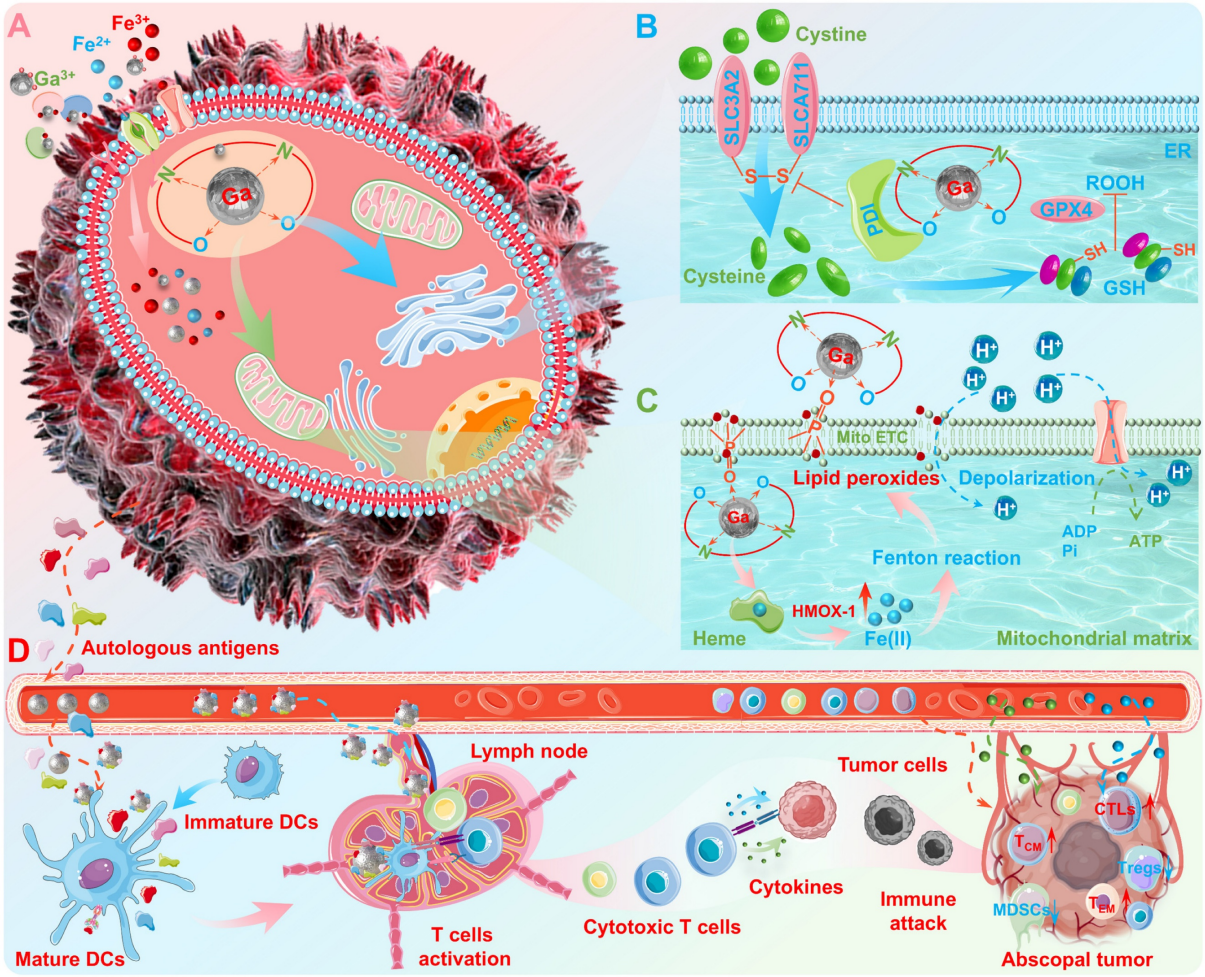
** The antineoplastic activity of gallium.** (A) "Iron competition" mechanism; (B) ER stress-mediated cell death, and enforced ferroptosis *via* preventing GSH repair; (C) Disruption of membrane integrity, triggering lipid peroxides and ultimately ferroptosis. (D) Antigen-capturing and immunostimulatory gallium nanoplatform for reconstructing the positive tumoricidal-immunity feedback loop. Created with Smart.Servier.com.

In light of the rapid metabolic and proliferative characteristics of cancer cells, their physiological processes require significantly more iron than healthy cells 65. Based on this biological difference, scholars have also explored the antineoplastic activity of gallium-based pharmaceuticals mainly relying on the "Trojan horse" strategy (Figure 3A) 16. This strategy precisely targets the weaknesses of cancer cells, laying a solid biochemical foundation for the development of gallium-based anti-cancer drugs. With the deepening of research, the field of gallium-based anticancer drugs is developing rapidly, and is expected to bring revolutionary breakthroughs in cancer treatment.

Although the "Trojan horse" strategy provided an initial insight into gallium's antineoplastic activity, as scientific research has progressed, researchers have come to realize that gallium-induced antineoplastic effects may be far more complex than a simple "iron competition" mechanism 66, 67. Previous studies revealed that gallium (in the form of compounds or complexes) can trigger the production of mitochondrial ROS, which in turn up-regulates the expression of metallothionein and heme oxygenase-1 (HMOX-1) 68, 69. However, the researchers have not directly associated these biological phenomena (consistent with redox homeostasis dysregulation) with "ferroptosis", since they were discovered before the concept of "ferroptosis" was introduced. Subsequently, the pioneering studies carried out by Kasparkova J. et al. 70 and Pettinari R. et al. 71 have revealed that the gallium complexes can induce ferroptosis *via* iron metabolism disruption or cellular redox homeostasis dysregulation. In addition, several studies have revealed the mechanism of gallium complex-induced ferroptosis. Particularly noteworthy, Zhang J. et al. reported a gallium complex (Ga^3+^ complex with planar salen ligands) that targets protein disulfide isomerase (PDI), which may induce endoplasmic reticulum (ER) stress-mediated cell death (Figure 3B) 66. While the subsequent mechanistic studies have shown that this gallium complex can act as an effective anion transporter, which may disrupt membrane integrity and trigger an overload of cellular iron ions, leading to the accumulation of lipid peroxides and ultimately ferroptosis (Figure 3C) 16. More importantly, they also found that this gallium complex may also target PDI located in the ER membrane, thus enforcing ferroptosis *via* preventing the repair of the antioxidant glutathione (GSH, which may inhibit ferroptosis) system (Figure 3B). Unlike the classical "iron competition" hypothesis, this mechanism of synergistically induced ferroptosis may provide important clues for elucidating the antineoplastic activity of gallium-based pharmaceuticals. Furthermore, previous studies have also demonstrated that gallium and its derivatives possess significant immunostimulatory capabilities in cancer immunotherapy, which promises to be another representative anticancer mechanis 43, 72, 73. For example, our group has revealed that gallium nanoplatform may not only effectively destroy orthotopic tumors (upon external energy stimulation) to generate multifarious autologous antigens, but may also enable precise modulation of antigen-presenting cells (APCs, primarily dendritic cells) functionality *via* immunoadjuvant capacity, which ultimately awaken robust and durable antineoplastic immune responses (Figure 3D) 43. The potential immunoadjuvant capacity of gallium nanoplatform may benefit from the unique adsorbability of the spontaneously formed gallium oxide surface layer ("depot effect" mechanism, enable sustained antigen release to prolong the bioavailability of immunogens) and intrinsic immunostimulatory properties of gallium (metallicity, to induce cytokines secretion, and enhance maturation/activation of APCs). While the antineoplastic immune responses may ascribe to enhanced differentiation and recruitment of T lymphocytes (e.g., tumor-specific cytotoxic T lymphocytes, central memory T cells and effector memory T cells), upregulated secretion of pro-inflammatory cytokines and chemokines cytokines secretion (e.g., interleukin 6 (IL-6), interleukin 12 (IL-12), tumor necrosis factor α (TNF-α), and interferon gamma (IFN-γ)) and remodeling of the immunosuppressive tumor microenvironment (e.g., immunosuppressive cells reduction). However, the interactions between gallium-based pharmaceuticals and organism and the antineoplastic mechanisms still deserve more in-depth elucidation, which is crucial for the design and development of novel gallium-based anticancer drugs.

### Osteogenic Activity

Gallium, as a unique metallic pharmaceutical element, has attracted widespread attention in the field of orthopedics, while its remarkable osteogenic activity provides a new perspective for the treatment of a variety of bone-related diseases 13, 74-78.

**Figure 4:**
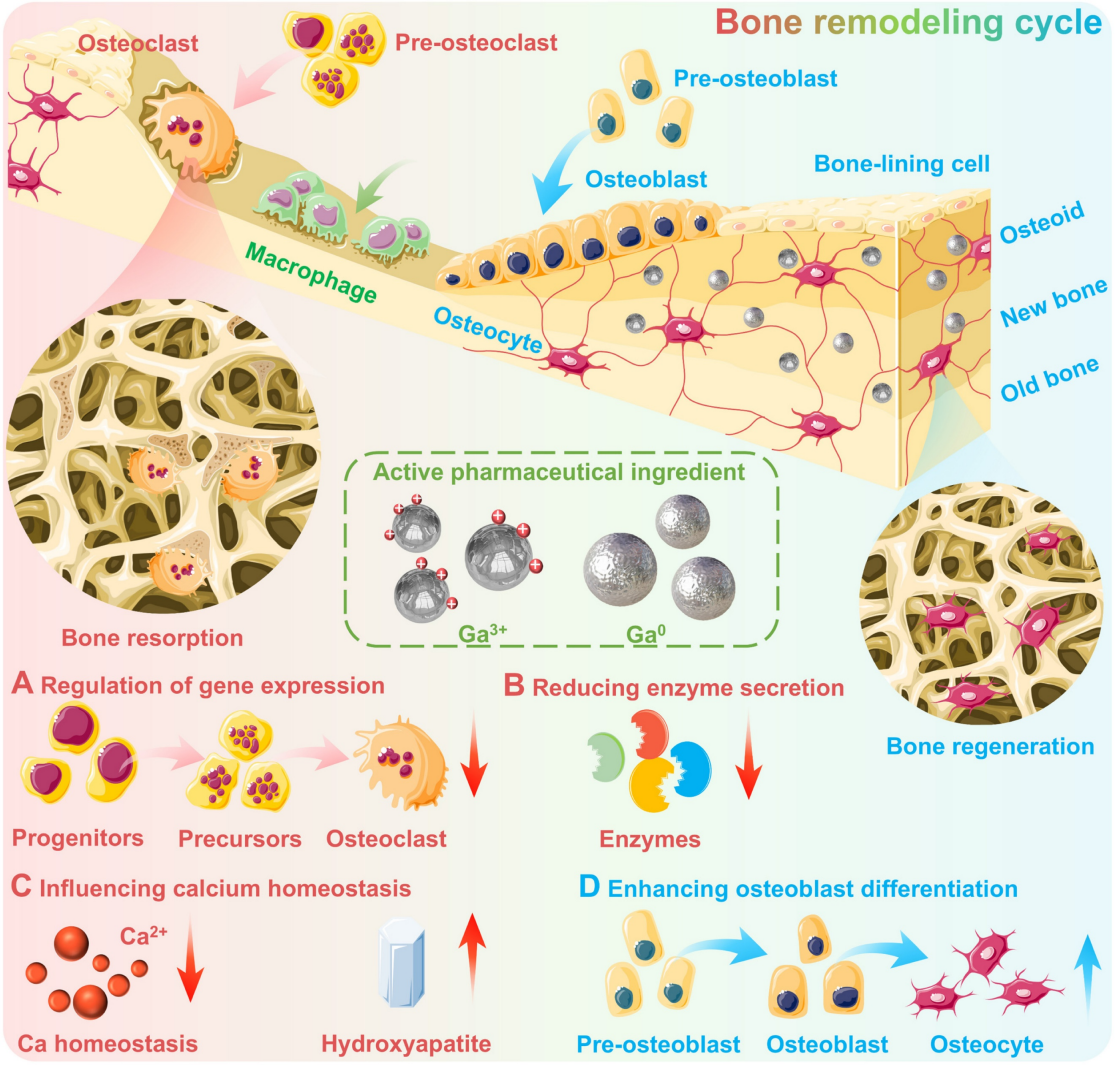
** The osteogenic activity of gallium.** (A) Regulation of gene expression: gallium may inhibit osteoclast differentiation and hence impede bone resorption; (B) Reducing enzyme secretion: gallium possesses inhibitory effects on enzymes responsible for bone matrix degradation and bone resorption; (C) Influencing calcium homeostasis: gallium may reduce bone destruction *via* affecting calcium homeostasis, and increase bone calcium content and hydroxyapatite crystallite perfection; (D) Enhancing osteoblast differentiation: gallium may promote bone regeneration *via* enhancing the early differentiation of osteoblasts phenotype. Created with Smart.Servier.com.

The osteogenic activity of gallium is primarily derived from a dose-dependent anti-osteoclastic effect, which may reduce the resorption, differentiation and formation of osteoclasts without negatively affecting osteoblasts 79. Since osteoclasts as multinuclear giant cells play an important role in releasing minerals and other molecules stored in the bone matrix, which are responsible for the decomposition and resorption of bone tissue. While osteoblasts are responsible for bone tissue remodelling (Figure 4) 80. The osteogenic mechanisms of gallium-based drugs are complex and subtle, mainly including the following aspects 79, 81, 82: 1) **Regulation of gene expression**: Gallium can regulate the expression of genes closely related to bone metabolism, thus contributing to maintaining bone stability (Figure 4A). For example, Ga can significantly downregulate the expression of specific osteoclast differentiation early marker genes, including NFATc1, FRA-2, JDP-2 and JUND, thereby inhibiting osteoclast differentiation 79, 82. And may also inhibit the osteocalcin gene expression and subsequently impede bone resorption, since the osteocalcin as osteoblast-specific bone matrix protein can trigger osteoclast resorption 81. 2) **Reducing enzyme secretion**: Gallium exhibits significant inhibitory effects on enzymes responsible for bone matrix degradation and bone resorption, such as matrix metalloproteinases (MMP13), which helps to maintain the integrity of the bone tissue (Figure 4B). 3) **Influencing calcium homeostasis**: Gallium may reduce bone destruction *via* affecting calcium homeostasis, e.g., blocking Ca²⁺ entry through TRPV-5A channels (a calcium channel highly expressed in osteoclasts) to interfere with the normal physiological function of osteoclasts 82. Additionally, gallium may also increase bone calcium content and hydroxyapatite crystallite perfection of bone tissue by affecting dissolution behavior (e.g., reducing acid secretion from osteoclasts) (Figure 4C) 75. 4) **Enhancing osteoblast differentiation:** It is noteworthy that gallium can preferentially accumulate in active bone-forming areas 83 and enhance the early differentiation of osteoblasts phenotype 78, 84, which further confirms its potential to promote bone regeneration (Figure 4D).

Overall, gallium-based pharmaceuticals hold great potential as novel orthopedic drugs in light of gallium's dual role in inhibiting bone resorption and promoting bone regeneration, which may bring about new therapeutic hope in the fields of osteoporosis, bone destructive diseases, and dental/orthopedic implants 7, 76, 80, 84-86. In the future, the application prospect of gallium-based orthopedic drugs will be more extensive.

## Gallium based Pharmaceuticals

Building on the foundational pharmaceutical activities of gallium, this section systematically explored the representative pharmaceutical formulations, such as compounds, complexes, nanomedicines, sensitizers and bioactive materials, in terms of practical applications for therapeutic agents, diagnostic agents, and drug carriers. In order to provide a quick and clear overview of the topic, the composition, structure, properties, applications, administration routes, *in vivo* dosage of representative pharmaceutical formulations are summarized in Table 1.

### Therapeutic agents

#### Gallium (III) compounds

**Figure 5:**
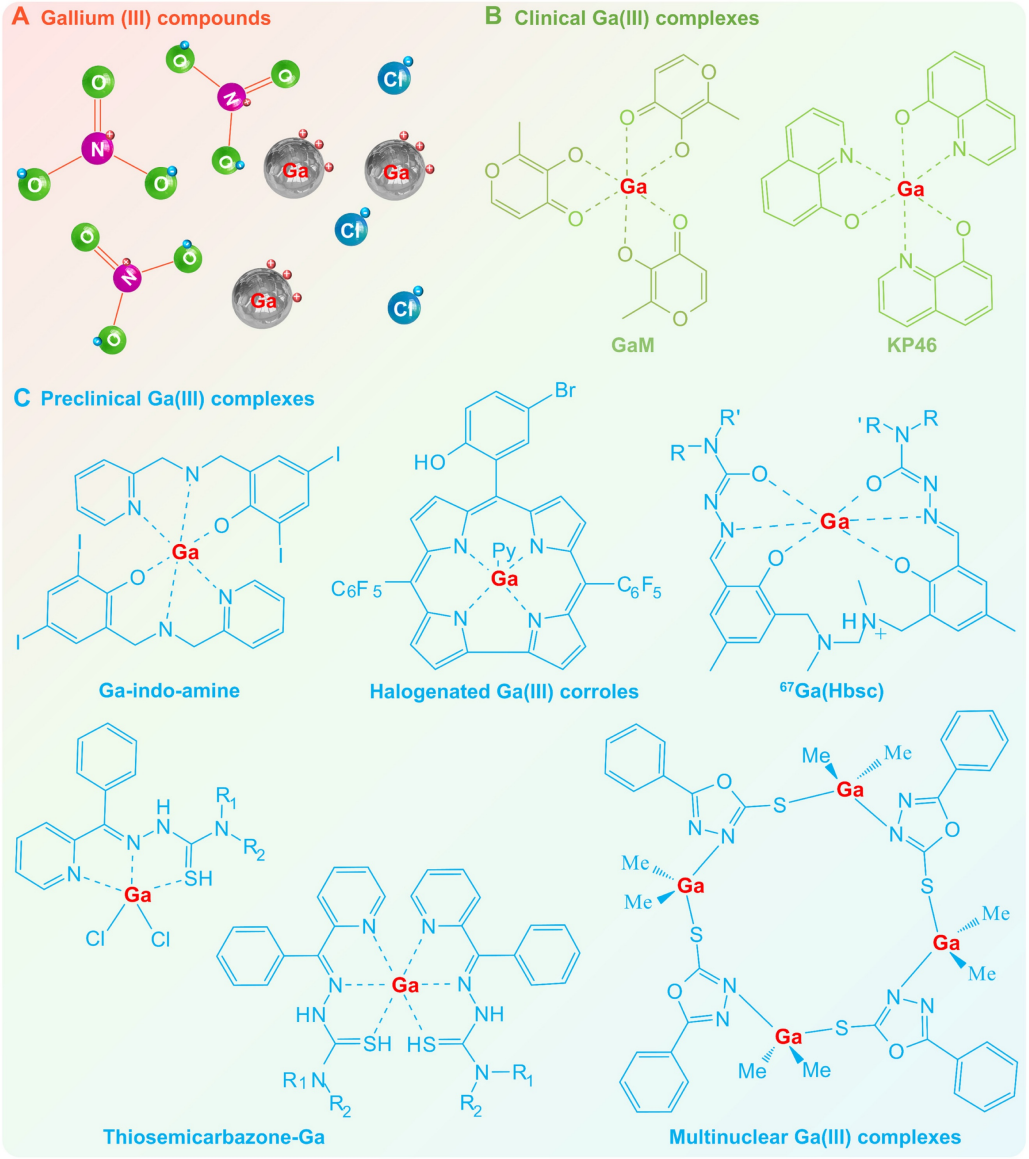
** Gallium (III) compounds and Gallium (III) complexes.** (A) Ga(III) compounds, e.g., gallium nitrate and gallium chloride, may completely ionize in aqueous solution and undergo hydrolysis, therefore posing challenges in terms of decreased bioavailability and pharmacological effects. The 2nd generation Ga(III) therapeutic agents: (B) Clinical Ga(III) complexes, e.g., gallium maltolate (GaM) and tris(8-quinolinolato)gallium(III) (KP46); (C) Preclinical Ga(III) complexes with diverse coordination configurations.

Due to the chemical reactivity, gallium tends to form trivalent ions (Ga^3+^) in compounds such as gallium nitrate (Ga(NO_3_)_3_) and gallium chloride (GaCl_3_), which have great potential for therapeutic applications (e.g., antibacterial, antitumor, and bone regeneration) 7, 10. Gallium nitrate was the first FDA-sanctioned gallium compound for the treatment of cancer-related hypercalcemia since the 1990s 13, and has subsequently been demonstrated to possess antineoplastic activity against human non-Hodgkin's lymphoma and advanced bladder cancer 106. While gallium chloride has also shown selective anticancer activity with an action mode similar to that of gallium nitrate (IC_50_, 100 μM ~ 1 mM) 16. Both gallium nitrate and gallium chloride may completely ionize in aqueous solution due to the weak coordination ability of nitrates and chlorides (Figure 5A), suggesting that the pharmacological activity of gallium compounds is derived from free Ga^3+^ ions and their hydrolysates 16. Current studies have shown that the therapeutic mechanisms of Ga^3+^ ions are thought to be closely related to the similarity with Fe^3+^ ions 6. Therefore, Ga^3+^ as a mimic of Fe^3+^ may disrupt fundamental biological processes that require the involvement of Fe^3+^, such as iron-catalyzed reactions, extracellular transport and cellular uptake in various biological systems, offering therapeutic opportunities 6, 17. It is worth mentioning that iron-dependent physiological processes are necessary for various types of living organisms, therefore gallium-based pharmaceuticals are less susceptible to drug resistance caused by target mutations, altered metabolic processes, signaling pathway reorganization, drug effect diminution, etc. 107, 108.

#### Gallium (III) complexes

The therapeutic effect of Ga (III) is significantly affected by its speciation, since the Ga (III) compounds may undergo hydrolysis when dissolved in water or saline (Figure 5A) 16. For example, the Ga^3+^ ion may hydrolyze to a hydroxide mixture of Ga(OH)_11_^8-^ and Ga(OH)_3_ at pH ~ 4; and Ga(III) hydroxides mixture of Ga(OH)_3_ and Ga(OH)_4_^-^ at physiological pH ~ 7.4 109. With the hydrolysis of Ga^3+^ ions, the drug solutions become highly acidic accompanied by a decrease in bioavailability and pharmacological efficacy, thereby affecting its therapeutic application 16. Hence, the Ga(III) complexes with ligand protection have emerged as 2^nd^ generation Ga(III) therapeutic agents, with the rationale of preventing hydrolysis through chelation of organic ligands, thereby enhancing bioavailability and reducing adverse effects (Figure 5B-C). As a prime example, gallium maltolate (GaM, tris(3-hydroxy-2-methyl-4*H*-pyran-4-onato)gallium 110, 111) and tris(8-quinolinolato)gallium(III) (KP46) 91 are the most representative candidates (Figure 5B). Through the complexation of organic ligands, the Ga^3+^ speciation in the physiological environment can be effectively regulated, thereby changing the cellular uptake mechanism and action mode, which largely overcomes the unfavorable pharmacokinetic and toxicological properties specific to gallium compounds 16, 91. Specifically, the antineoplastic activity of GaM and KP46 is greatly enhanced against different cell lines (IC_50_ 10 ~ 40 μM for GaM^90^, and 1 ~ 3 μM for KP46 91, respectively), while the biosafety is also greatly improved. In addition, gallium complexes possess better kinetic stability and gastrointestinal absorption, thus allowing for oral administration in smaller doses, whereas gallium compounds are only suitable for intravenous administration 112. Encouraged by the clinical trial results of GaM and KP46, novel Ga(III) complexes with different coordination configurations are emerging and show great potential for further medicine development (Figure 5C) 16, 66, 67, 71, 113, 114. Since Ga(III) may bind to virtually any complex that can bind Fe(III), simple iron chelators as well as more complex siderophores and hemes are potential carriers to increase bioavailability of Ga(III) 115. For example, 1) iron chelators, including quinolinolato, naphthoquinone, desferrioxamine B, protoporphyrin IX, deuteroporphyrin, chlorin e6, mesoporphyrin, hematoporphyrin, octaethylporphyrin and porphine; 2) siderophores and heme, including acinetoferrin, staphyloferrin A, cepacianchelin, and dihydroxybenzoyl-serine; all of which can form therapeutic complexes with Ga(III) 7, 115.

#### Gallium-based nanomedicine

In addition to restricting the hydrolysis of the active pharmaceutical ingredient (Ga^3+^ ions) by chelation or coordination, it is also possible to enhance bioavailability and impart additional therapeutic functionality by constructing Ga(0)/Ga(III)-based nanomedicines.

**Figure 6:**
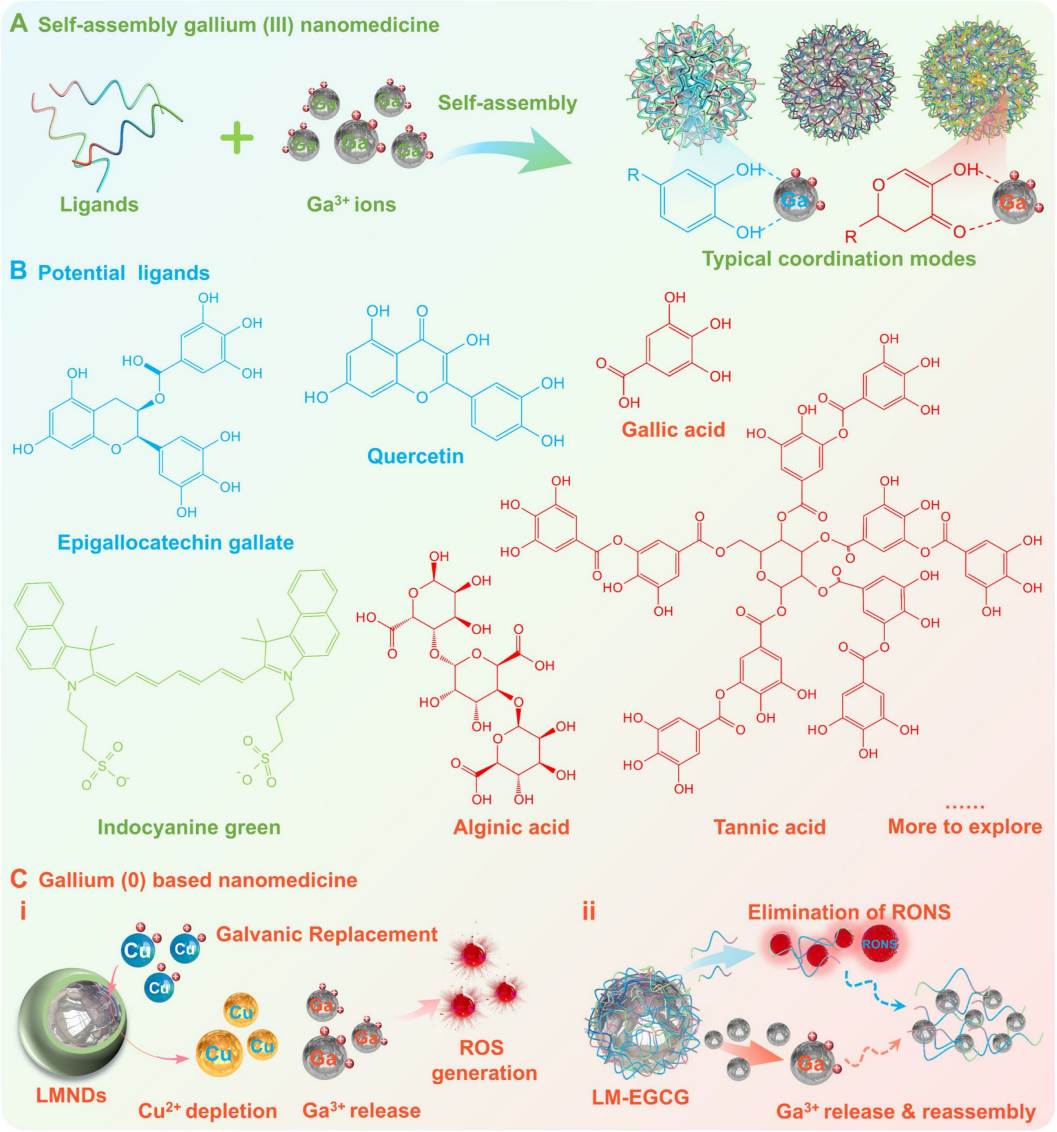
** Gallium-based nanomedicine.** (A) Gallium (III) based nanomedicines synthesized by self-assembly of Ga^3+^ ions with ligands. (B) Potential ligands including polyphenols, flavone, aldehyde acid, and indoles. (C) Gallium (0) based nanomedicine utilizing the intrinsic therapeutic activity of gallium-based LMs and released active pharmaceutical ingredients (Ga^3+^ ions).

Fundamentally, it is a facile and efficient approach to form nanomedicines by self-assembly of Ga^3+^ ions with ligands (e.g., polyphenols (gallic acid, tannin acid, alginic acid), flavone (chrysin, quercetin, epigallocatechin gallate), aldehyde acid (alginic acid), and indoles) (Figure 6A-B) 23-25, 73, 116. The morphology and dimensions of Ga^3+^ based nanomedicines can be designed by modulating buffer type, pH, reaction time, precursor concentration and coordination mode. While the additional therapeutic functionality can be enhanced or endowed utilizing the natural functionality of the organics (e.g., antibacterial, anti-inflammatory, and anticancer effects) as well as the functional components (e.g., enzymes, drugs) loaded on the Ga^3+^ based nanomedicines, thus realizing the potential for a variety of biomedical applications. For example, Min Zhou et al. synthesized ultrasmall non-antibiotic gallium-based nanomedicine by a facile one-step process using Ga^3+^ ions and hepatic-targeted indocyanine green molecules, which may eradicate multi-drug-resistant (MDR) bacteria and disrupt biofilm *via* the synergistic effect of photodynamic therapy and iron metabolism blockade, therefore significantly improved the treatment outcomes of infected liver abscesses and keratitis 23. In another noteworthy cutting-edge advancement, Xian-Zheng Zhang's group designed an inhalable microbial capsular polysaccharide-camouflaged gallium-polyphenol metal-organic network, which can be used as a dual-acting nanomedicine to overcome microbial-induced chemoresistance by eliminating local microbial in lung cancer 25.

Additionally, researchers have also begun to explore the intrinsic therapeutic activity of Ga-based liquid metals, to avoid the delicate and cumbersome process of screening and synthesizing pre-drugs, simplify the therapeutic strategy and enhance the selective therapeutic effect. As early as 2018, Hyung-Jun Koo's group has demonstrated that under aqueous conditions, only Ga³⁺ ions are dominantly released from Ga-based liquid metals (e.g., gallium-indium eutectic alloy), laying the critical mechanism for the sustained release of the active pharmaceutical ingredient (Ga³⁺ ions) while avoiding hydrolysis and enhancing bioavailability 37. Building on this foundation, Zhen Gu's group recently discovered that small-sized (~20 nm) gallium-based liquid metal nanodroplets (LMNDs) can be used as a nanomedicine with excellent tumor permeability and biocompatibility, which can achieve the conversion of 0-valent Ga to Ga^3+^ ions by electrochemical substitution depletion of Cu^2+^ ions in cancer cells, accompanied by a large amount of reactive oxygen species (ROS) generation, thus leading to selective apoptosis and anti-angiogenesis in breast cancer cells (Figure 6C (i)) 26. This *in vivo* nanocarrier-to-nanomedicine conversion strategy not only directly and effectively avoids hydrolysis of the active pharmaceutical ingredient (Ga^3+^ ions), but also explores a novel pharmaceutical formulation. As another representative research advance, Lin Wang and Zheng Wang's team co-proposed an orally administered gallium-based liquid metal nanodrugs, i.e. epigallocatechin gallate (EGCG) encapsulated gallium-indium eutectic alloy with nano-sized formulations (LM-EGCG) 27. After oral administration and degradation, the dissociative EGCG with favorable adhesion activity and coordination ability, could not only adhere to electropositive inflamed tissue for effective elimination of reactive oxygen and nitrogen species (RONS), but also capture dissociated Ga^3+^ ions to form metal-polyphenol complex for modulation of the dysregulated microbiome (Figure 6C (ii)). Based on the amplification-targeting strategy, this nano-sized formulation can effectively alleviate inflammatory bowel disease while avoiding low bioavailability and systemic adverse effects caused by rapid clearance and off-target effects.

#### Gallium-based liquid metal sensitizers

In the biomedical domains, the intrinsic energy-responsive properties of gallium-based LMs can be used to achieve external-field energy conversion with accompanying changes in physical and chemical properties, implying that gallium-based LMs can be applied as energy sensitizers for physicochemical therapies 117.

**Figure 7:**
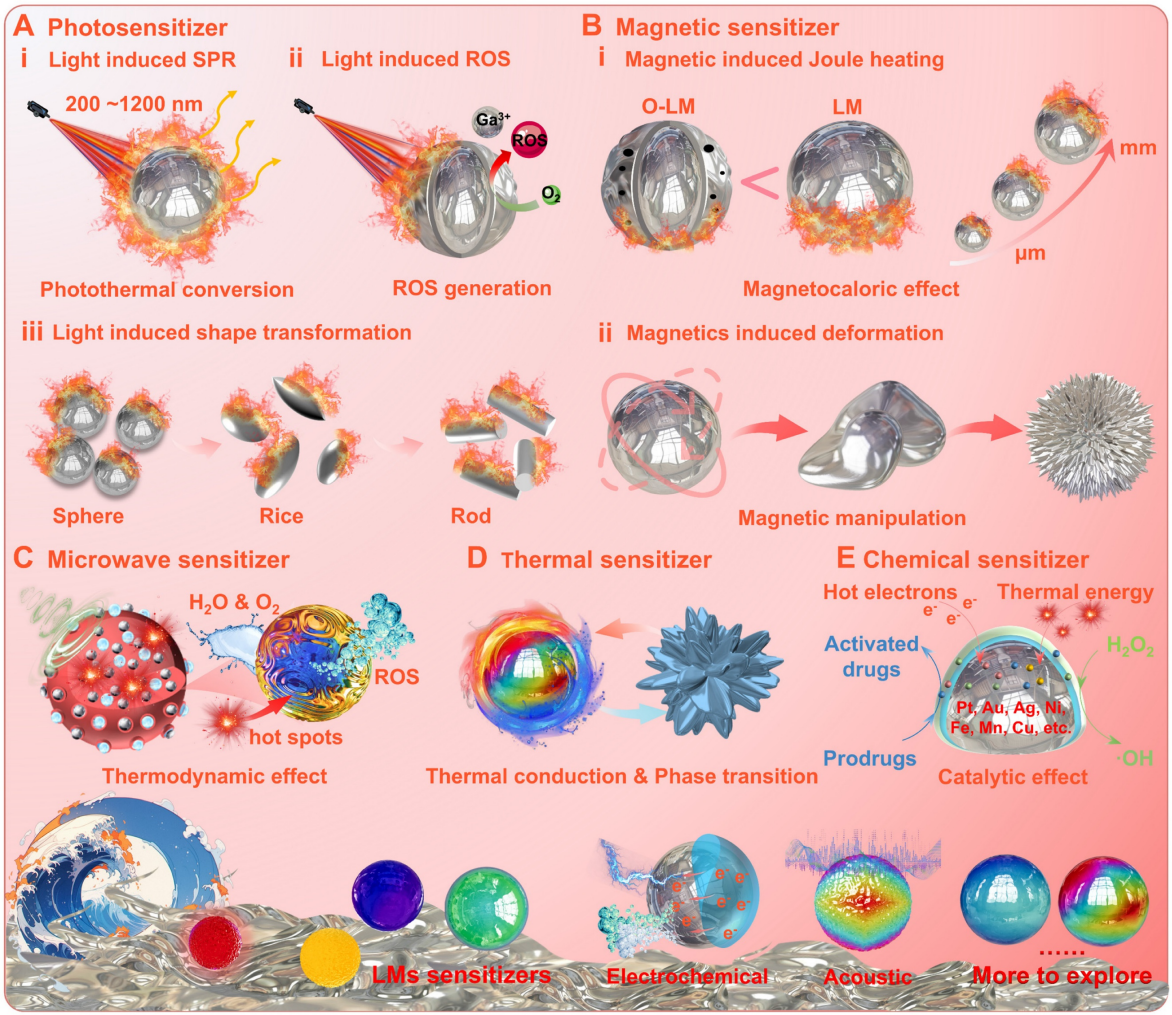
** The gallium-based liquid metals act as energy sensitizers for physicochemical therapies:** (A) Photosensitizer; (B) Magnetic sensitizer; (C) Microwave sensitizer; (D) Thermal sensitizer; (E) Chemical sensitizer.

The photosensitive conversion property is one of the most widely studied. The photo-response mechanism studies have shown that part of the light is absorbed by gallium-based liquid metal nanoparticles (LMNPs), while the remaining part is reflected or re-emitted at the same/shifted frequency 118-122. The plasma frequency (i.e., electron cloud oscillation frequency) of LMNPs lies in the energy range comparable to that of ultraviolet (UV) light 123, 124 and can be manipulated by dimensional, structural, and morphological adjustments to achieve a shift of the resonance peak from the UV region to the visible region 124, 125. When the oscillation frequency of the LMNPs matches the frequency of the incident photons, the localized surface plasmon resonance (LSPR) phenomenon may arise due to the strong absorption effect (Figure 7A (i)) 124, 126. Thus, the LMNPs show similar capabilities to absorb UV, visible and near-infrared light, as photosensitizers such as carbon-based materials 92 and melanin 127. Benefiting from the LSPR effect and chemical activity, the LMNPs may generate a large amount of heat and reactive oxygen species (ROS) (Figure 7A (ii)), and may undergo photo-triggered deformation under light irradiation at specific wavelengths (Figure 7A (iii)) 33. Basically, the photothermal phenomenon is caused by the dissipation of the absorbed light energy into heat (with the calculated photothermal conversion efficiency ranges from 25.3 % to 53.0 %) 117. While the ROS generation and photo-triggered deformation are attributed to electrochemical reactions triggered by thermal energy and the ambient environment. Specifically, the ROS generation is driven by electron transfer from Ga to the water and oxygen: Ga - 3e = Ga^3+^, O_2_ + e = ·O_2_, ·O_2_ + 2H_2_O + 3e = 4·OH; And the photo-triggered deformation is induced by the heat-induced oxidation of Ga into the intermediate crystal product (GaOOH): 4Ga + 3 O_2_ + 2H_2_O → 4GaOOH, 2Ga + 2H_2_O → 2GaOOH + 3H_2_ ↑.

After the photosensitive properties were uncovered, researchers have also explored a variety of energy-responsive properties of gallium-based LMs with applications in disease treatment (for details, see Ref 117). For example, under alternating magnetic fields (AMF), the gallium-based LMs may undergo remarkable magnetocaloric phenomenon and magnetics-induced deformation, positioning them as promising candidates for magnetic-mediated hyperthermia and controlled drug release (Figure 7B) 128-132. Unlike conventional ferromagnetic materials (e.g., Fe₃O₄ nanoparticles), which rely on Néel and Brownian relaxation mechanisms requiring high-power AMF, the magnetocaloric effect of gallium-based LMs primarily arises from eddy currents (lead to substantial Joule heating) induced by electromagnetic induction (Faraday's law, due to favorable electrical conductivity, e.g., GaIn_24.5_, EGaIn 3.4×10^6^ S/m) 128-133. Notably, the magnetocaloric effect of gallium-based LMs is closely correlated with oxidation degree and dimensions, since the surface oxide layers and corroded holes may degrade electrical conductivity while reduced dimensions may impair eddy current-induced thermal effect (Figure 7B (i) 131. Meanwhile, as conductive fluids, the gallium-based LMs may also be manipulated by the Lorentz force under AMF 128, while the magnetics-induced deformation may be significantly enhanced (with sharper morphological edges) when doped with magnetic particles (Figure 7B (ii)) 56. Besides, under microwave (MW) radiation, LMNPs can also effectively utilize the hot spots triggered by MW energy to achieve free radical generation (ROS) (Figure 7C) 94, 134. In addition, phase transition solidification can also promote dramatic shape transformation of gallium-based LMs, mainly due to solidification induced volume expansion (Figure 7D) 95, 101, 135. For example, a spherical to cactus-like structure transformation of gallium particles can be achieved upon freeze-solidification, since liquid gallium with a density of 6.095 g/mL solidifies into an α-Ga phase with a density of 5.904 g/mL 95. More importantly, beneficial from the fluidic nature and electron-rich environment, gallium-based LMs may also facilitate catalytic reactions (e.g., bioorthogonal catalysis or Fenton-like catalysis) to achieve external stimuli enhanced catalytic therapy (Figure 7E) 41, 136. Fundamentally, the inherent capability of monophasic gallium-based LMs (molten state) to accommodate additional trace metallic elements (e.g., Pt, Au, Ag, Ni, Fe, Mn and Cu) 123, enables the design of highly efficient liquid catalyst systems 96, 137-143. For instance, Kourosh Kalantar-Zadeh's group reported that trace amounts of Pt (exist in liquid form) can spontaneously dissolve in liquid Ga without atomic segregation, effectively activating adjacent Ga atoms for catalysis 137. Under physiological conditions, these liquid catalyst systems may actuate catalytic reactions with superior catalytic performance, such as converting tumor endogenous H₂O₂ into ROS 142 and activating prodrugs (Figure 7E) 96. While the generation of hot electrons and thermal energy under external stimuli (e.g., light), may further synergistically amplify catalytic efficiency, ultimately achieving exceptional therapeutic efficacy (Figure 7E) 96, 142-144.

Briefly, upon the interaction with external energy sources and ambient environment, the gallium-based LMs act as energy sensitizers can convert the external energy (e.g., optical, magnetic, electrical, acoustic, chemical, etc.) into orther forms (e.g., localized hyperthermia, ROS generation, chemical reactions or sharp physical deformations, etc.) within the lesion area 30, 117. Therefore, it is expected to achieve a variety of therapeutic modes, such as targeted thermotherapy, dynamic therapy, mechanical therapy, chemotherapy or catalytic therapy, which is promising in biomedical fields (e.g., antimicrobial and antineoplastic therapy).

#### Gallium-containing bioactive materials

**Figure 8:**
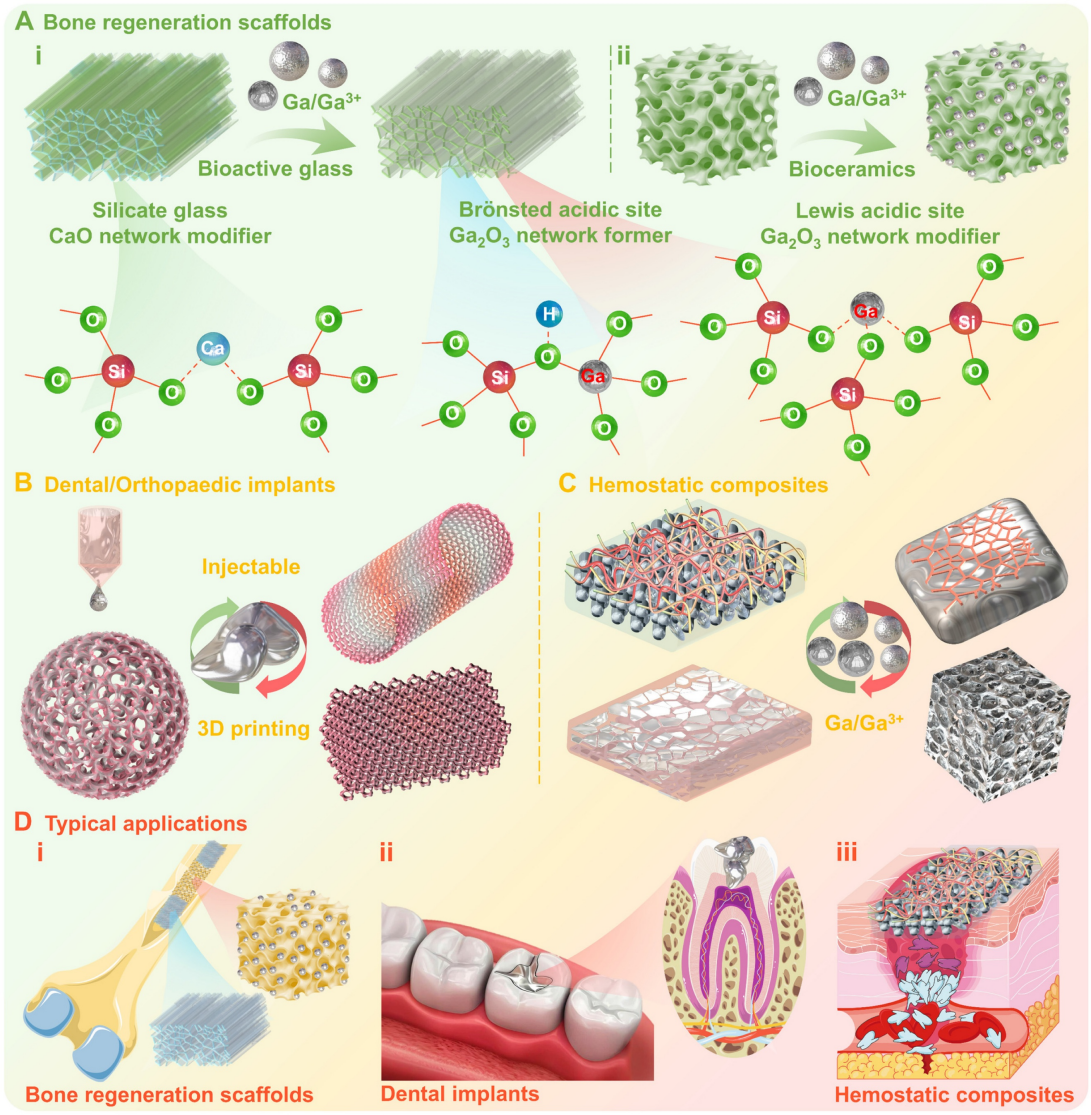
** Gallium-containing bioactive materials.** (A) Bone regeneration scaffolds, the gallium incorporation within i) bioactive glasses and ii) bioceramics may act not only as network former/modifier, but also as active pharmaceutical ingredients; (B) Dental/Orthopaedic implants, the gallium incorporation may induce antimicrobial effects without affecting biocompatibility and mechanical properties, and also offer imaginative scope for injectable/3D printing implants; (C) Hemostatic composites, gallium-containing bioactive composites may alleviate inflammatory response (antimicrobial and anti-inflammatory activity) and trigger haemostasis; (D) Schematic diagram of typical applications. Created with Smart.Servier.com.

Although gallium has exhibited significant therapeutic activities, including antibacterial, anti-inflammatory, anticancer, and osteogenic activities, its ideal administration modality still needs to be further optimized. Currently, gallium-based pharmaceuticals can be administered by a variety of routes, such as oral administration, transdermal administration, inhalation administration, gastrointestinal administration, and injection administration. However, conventional gallium-based pharmaceuticals in the form of salts or complexes have a low effective gallium dose within the target lesion when administered orally, and require prolonged and continuous infusion when administered intravenously, which poses an inconvenience in practical applications. As an innovative alternative, gallium-containing bioactive materials that deliver active pharmaceutical ingredient Ga(0)/Ga(III), exhibit great potential for widespread clinial applications. However, this area has not yet been extensively explored.^80^ Wherein, the incorporation of Ga(0)/Ga(III) as a dopant into various bioactive material types, such as scaffolds 145, hydrogel 146, fibers 147, bioactive glasses 84, bioceramics 148, and composites 149, may induce multiple therapeutic effects and provide unique properties (Figure 8).

1) Bone regeneration scaffolds: Bioactive glasses possess favorable biocompatibility, bioactivity, and biodegradability, which can rapidly form a bioactive layer with bone growth and regeneration ability after contacting with biological tissues and can also be gradually degraded and absorbed *in vivo* 80. The gallium incorporation within glass structures, may act both as network former and network modifier (similarly to Al^3+^) 150, since gallium may incorporate into tetrahedral (GaO_4_) and octahedral (GaO_6_) structural units (Figure 8A (i)) 84. Therefore, gallium incorporation may exert a significant influence on both structural and thermal performances of bioactive glasses. Bioceramics are a class of microcrystalline ceramic materials composed of dense hydroxyapatite, alumina, zirconia, or calcium phosphate, which may offer superior biocompatibility, mechanical compatibility, antithrombotic, sterilizability, and physicochemical stability 148. Studies have indicated that gallium may exist as an interstitial solid solution in hydroxyapatite crystals, without displacing calcium within crystals or causing matrix framework distortion (Figure 8A (ii)) 97. It is worth noting that gallium incorporation acts not only as a structural modifier, but also as an active pharmaceutical ingredient (e.g., antibacterial, antifungal, anti-inflammatory, antineoplastic or osteogenic agents). Meanwhile, the bioactive materials as matrix enable the controlled and precise delivery of gallium to the desired site, thereby increasing therapeutic efficacy and minimizing side effects 76, 84, 86, 148, 151. For instance, when gallium containing bioactive glass or bioceramics (as bone regeneration scaffolds) are filled in cavities formed after bone tumor surgical procedures, the gallium release may not only prevent the further growth and proliferation of cancerous cells (anticancer activity), but also enhance bone reconstruction (osteogenic activity) involving the inhibition of osteoblast differentiation, the enhancement of early differentiation of osteoclasts, and an increase in calcium and phosphorus content of the bone (Figure 8D (i)) 80.

2) Dental/Orthopaedic implants: Metals and alloys are widely used as dental/orthopaedic implants in clinical practice (Figure 8D (ii)), but sometimes may suffer the risk of complications due to bacterial infections 152. One of the feasible strategies for preventing bacterial infections is the formation of alloy biomaterials with antimicrobial metals, such as Ag, Cu and Zn 153. Previous comparative studies have shown that gallium-based liquid metal (gallium-indium eutectic alloy) could exhibit enhanced antimicrobial activity compared to gallium nitrate, owing to the synergistic antimicrobial effect originating from sustained gallium ions release 37 (avoiding hydrolysis, higher bioavailability) accompanied by ROS generation 54. In addition, the incorporation of small amounts of gallium (0.1-2 wt %) into titanium or magnesium alloys can also be effective in inducing antimicrobial effects against bacterial and fungal strains without affecting biocompatibility and mechanical properties 98, 154. More importantly, gallium-based LMs, as low melting point alloys, offer a wide imaginative scope for injectable/3D printing dental/orthopedic implants, due to the liquid-solid phase transition that can be conveniently achieved near body temperature (Figure 8B) 30, 32, 76, 86, 155.

3) Hemostatic composites: Hemostatic biomaterials are crucial in promoting wound healing, as they create favorable conditions for wound repair *via* rapid bleeding control. However, one of the major challenges is how to effectively prevent and control bacterial infections at the wound site, which may interfere with the healing process or even cause serious complications 156. Gallium-containing bioactive materials also show potential for wound healing, not only in the later stages *via* alleviating the inflammatory response (antimicrobial and anti-inflammatory activity), but also in the early stages *via* activating intrinsic coagulation pathways to trigger haemostasis (coagulation, platelet activation or thrombosis) (Figure 8C) 157, 158. For example, Pourshahrestani et al. reported a gallium-containing mesoporous bioactive glass/chitosan composite scaffold, where gallium participation effectively improved the hemostatic properties of matrix materials, showing higher thrombosis, coagulant activity and increased platelet adhesion (Figure 8D (iii)) 99.

Hence, gallium can be used to improve the properties of a wide range of bioactive materials and to confer additional therapeutic activities, which are attractive in typical medical applications such as cancer treatment, bone defect repair, soft tissue wound healing, and so on.

### Diagnostic agents

**Figure 9:**
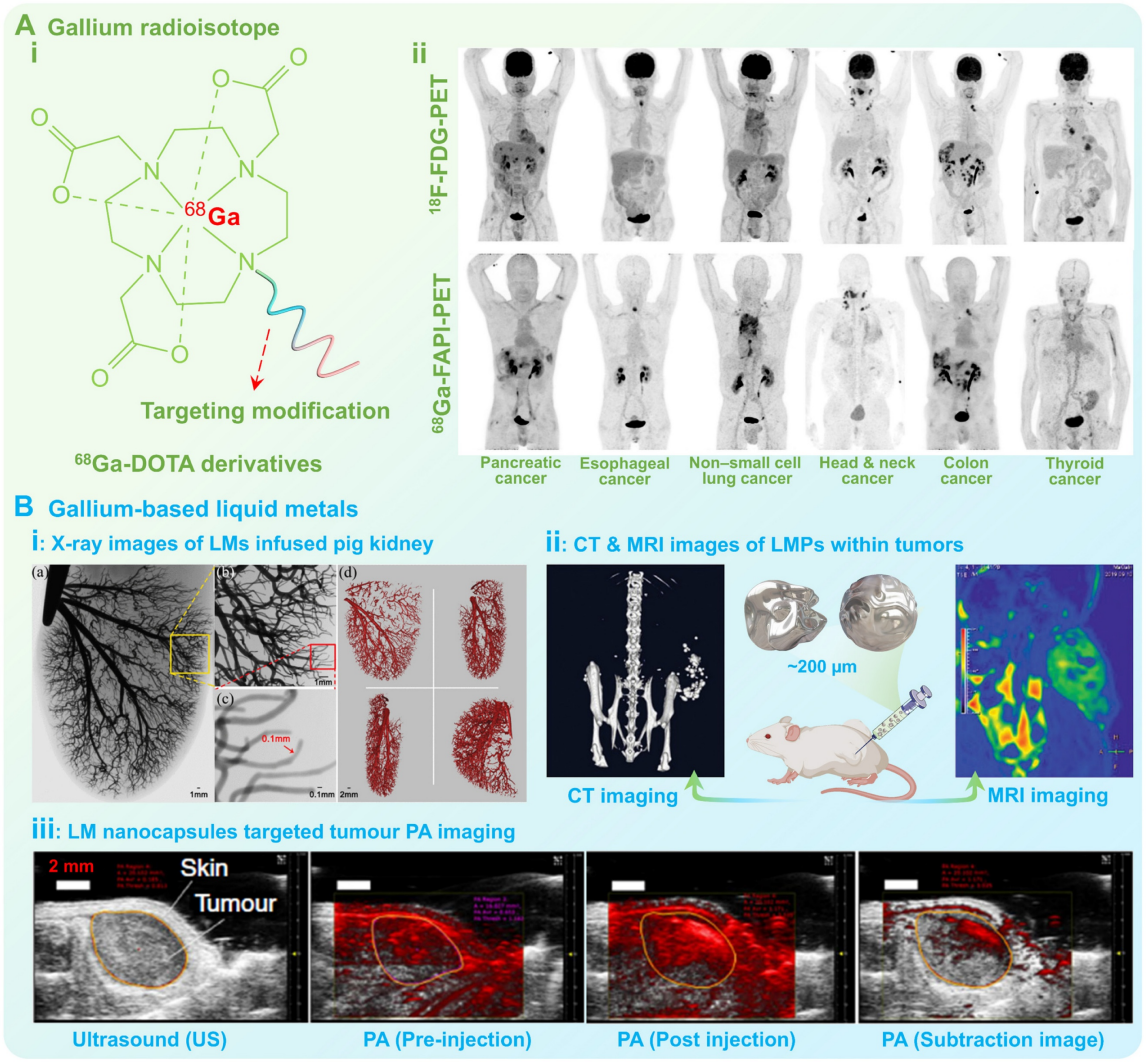
** Medical imaging of gallium radioisotopes and gallium-based liquid metals.** (A) Gallium radioisotopes as PET imaging contrast agents: i) Molecular structure of ^68^Ga-DOTA derivatives; ii) Intraindividual comparison of ^18^F-FDG PET and ^68^Ga-FAPI PET imaging in six patients with different tumor entities, conducted within less than 9 days. Reproduced with permission 22. Copyright 2019, Society of Nuclear Medicine and Molecular Imaging. (B) Gallium-based LMs for developing diagnostic agents: i) X-ray images of macroscopic liquid metals infused pig kidney; Reproduced with permission 22, 100. Copyright 2014, IEEE. ii) CT and MRI images of liquid metal particles within tumors; Reproduced with permission 101. Copyright 2020, Wiley-VCH; iii) Liquid metal nanocapsules for targeted tumor PA imaging; Reproduced with permission 33. Copyright 2017, Springer Nature.

The visualization of metallic pharmaceuticals is of great significance for modern medical science, since they can assist diagnosis of diseases, assessment of physiological conditions, supervision of *in vivo* drug distribution, evaluation of metabolism, and judgement of therapeutic prognosis. Owing to the intrinsic high density, electromagnetic properties, and radioactivity, the gallium radioisotopes (^67^Ga^3+^, ^68^Ga^3+^) and gallium-based LMs can be used as effective diagnostic agents in conventional medical imaging techniques (Figure 9), including X-rays 100, computed tomography (CT) 100, magnetic resonance imaging (MRI) 159, diagnostic radiography 160, positron emission tomography (PET) 20, and photoacoustic (PA) imaging 33, etc.

As a typical clinical radiographic agent, the gallium radioisotope ^67^Ga^3+^ can accumulate at the tumor site and trap electrons during the decay process to release gamma (γ) rays, thus has been permitted for the staging and diagnosis of clinical lymphomas 160. While another gallium radioisotope ^68^Ga^3+^ possesses a shorter half-life (68 min) and higher positron decay rate (89%), which has been applied as PET imaging contrast agents 20. In recent years, various diagnostic agents with diverse functions have been developed based on ^68^Ga(III)-DOTA and targeting small molecules or peptides (Figure 9A (i)), such as ^68^Ga(III)-DOTATOC, ^68^Ga(III)-DOTATE (targeting growth inhibitory receptor), ^68^Ga(III)-PSMA (targeting prostate-specific membrane antigen), and ^68^Ga-FAPIs (targeting fibroblast activating proteins), which can selectively target to the positive lesions for more efficient targeted diagnosis, thus have been widely used in the early diagnosis and preoperative staging of various types of malignant cancer (Figure 9A (ii)) 21, 22.

However, these gallium radiographic agents may separate from the chelating agents, and will be free within the body's circulatory system, thereby exhibiting varying degrees of acute toxicity to organs such as the kidney, liver and brain 5. To this end, researchers have attempted to develop a new generation of diagnostic agents utilizing gallium-based LMs with better biocompatibility and degradability than traditional gallium compounds/complexes (Figure 9B). For example, when radiopaque gallium-based LMs are injected into blood vessels, multi-scale vascular X-ray images can be obtained with very high contrast and enhanced penetration depth (Figure 9B (i)), suggesting that macroscopic gallium-based LMs can be used for high-definition X-ray imaging of capillaries (~100 µm) 100. Besides, gallium-based LMs also displayed favorable CT contrast enhancement when the size decreased to the micro-nanometer scale (Figure 9B (ii)) 44. In addition, gallium-based LMs also show promising T2-weighted MRI contrast-negative enhancement due to excellent electromagnetic properties (Figure 9B (ii)) 101. Moreover, nanosized gallium-based LMs are capable of strongly absorbing photon energy and undergoing localized surface plasmon resonance (LSPR) under light irradiation at a certain frequency 124, 126, which allows for specific optical applications. For example, gallium-based LMs can hybridize to produce strong hot spots and simultaneous fluorescence relying on the robust LSPR effect, providing an accurate and sensitive technique for biomolecules detection 102, 161. While the gallium-based LMs have also been recognized as promising photoacoustic couplers benefiting from the low attenuation and high acoustic impedance (17.4 MRayl, conferred by the liquid nature), thus can effectively enhance photoacoustic (PA) signals at the lesion site for clear PA imaging (Figure 9B (iii)) 33, 162.

Therefore, we can conclude that gallium radioisotopes and gallium-based LMs hold great promise for various diagnostic agents. As the research progresses, they will bring remarkable breakthrough in multi-mode medical imaging technology.

### Gallium-based drug carrier

Targeted drug delivery technology has attracted a lot of attention, which enables site-specific targeted drug delivery, showing the great potential for multimodal therapy. Gallium-based drug carriers are a promising class of drug carriers that can offer not only basic drug delivery functions, but also therapeutic activities in the form of hyperthermia, ROS generation, galvanic replacement etc 163.

**Figure 10:**
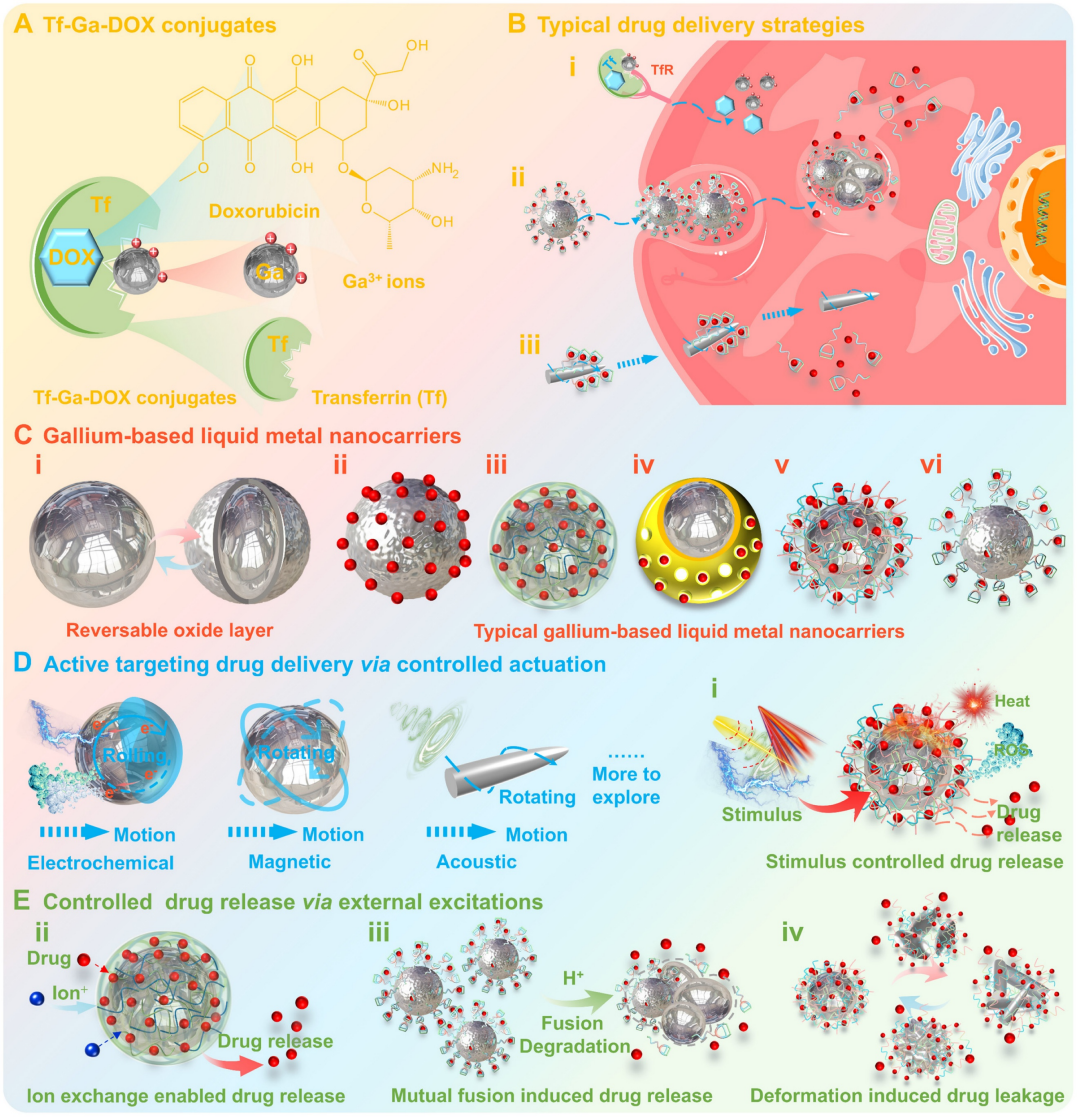
** Gallium-based drug carriers.** (A) Schematic structure of Tf-Ga-DOX conjugates. (B) Typical drug delivery strategies: i) Antibody-receptor-specific binding for active targeting drug delivery; ii) Drug delivery *via* endocytosis; iii) Controlled actuation for active targeting drug delivery; Created with Smart.Servier.com; (C) The surface modification for constructing gallium-based liquid metal nanocarriers: i) Reversible oxide layer formation on the surface; ii-vi) Diverse surface-modified gallium-based liquid metal nanocarriers, including simple surface oxidation, inorganic metal/non-metal structures, chemical, natural, and biological modification, may enhance drug-carrying capacity *via* physical/chemical adsorption. (D) Schematic diagram of active targeting drug delivery through controlled actuation driven by external stimulus. (E) Representative strategy for controlled drug release *via* external excitations.

As a typical example, gallium nitrate coupled to transferrin (Tf) and doxorubicin (DOX) (Tf-Ga-DOX conjugates, Figure 10A) can effectively reverse drug resistance after administration 9, 103, 164, 165. Specifically, after treating the multidrug-resistant MCF-7 cell line with Tf-Ga-DOX conjugates, the drug resistance has been overcome since multidrug resistance protein expression was decreased and the IC_50_ was reduced by approximately 100-fold compared to free DOX 103. The remarkable resistance reversal is attributed to the penetration of Tf-Ga-DOX conjugates into the cell *via* Tf-receptor-mediated transmembrane transport mechanisms (Figure 10B (i)) and inhibition of MRP gene expression. The conjugation of Tf with gallium shields the cell from recognizing DOX, thereby promoting drug accumulation in resistant cell nucleus and inducing cell death 165. While the Ga^3+^ self-assembled nanomedicines described in the previous section (**Gallium-based nanomedicine**) could also enable effective drug loading, thus enriching therapeutic modalities and effectiveness.

In addition, the additional scope could be provided for the targeted drug delivery by creating gallium-based LMs nanocarriers with highly tunable properties (Figure 10C) 17, 30, 117. Typically, the atomic Ga oxide layer (Ga_2_O_3_, thickness 0.7~3 nm 166, 167) may form at the gallium-based LMs-ambient environment interface 17, 168, which is partially passivating (similar to protective aluminum oxide) and may offer a barrier to prevent further oxidation of the gallium-based LMs (Figure 10C (i)) 17. However, the surface oxide layer could be reversed in response to external excitations (e.g., chemical, electrical, mechanical, temperature and pressure, etc.), leading to density, compositional, and structural changes (e.g., sustained oxidation, oxide rupture, dealloying and coalescence, etc.), which in turn may cause irreversible deterioration in properties and stability of the gallium-based LMs (Figure 10C (i)) 17. Currently, the additional surface modification is the most feasible strategy to improve the stability of gallium-based LMs nanocarriers, which could also endow them with diverse structures (e.g., microgel, capsule, heterophase, and core-shell) and preferable functionalities (e.g., drug-loading capacity, aqueous solubility, colloidal stability, biosafety, and stimulus-response properties) (Figure 10C (ii-iv)) 30. The surface modification of gallium-based LMs nanocarriers can be achieved by chemisorption, physical adhesion, electrostatic adsorption, ligand assembly, polymerization, bioconjugation and galvanic replacement 40. While the substrates that have been employed for the construction of surface-modified gallium-based LMs nanocarriers include inorganic and organic materials, such as organic ligands 169, polysaccharides 170, biomolecules 72, 95, and metal/non-metal materials 93, 171, 172, etc.

Basically, these surface modifiers as drug-carrying substrates can enhance the drug-carrying capacity of gallium-based LMs nanocarriers through physical/chemical adsorption, including hydrogels with three-dimensional network structure (Figure 10C (iii)) 44, 45, silica with porous structure (Figure 10C (iv)) 93, ligands with polycyclic structure (Figure 10C (vi)) 173, etc. Meanwhile, unlike conventional “static'' or “rigid” inorganic nanocarriers, gallium-based LMs nanocarriers possess distinctive transformability and stimuli-sensitive properties, which may effectively enhance bioavailability through coordinated drug delivery and controlled release. Compared to conventional nanocarriers that rely on passive targeting (EPR effect and endocytosis, Figure 10B (ii)) or active targeting (antibody-receptor specific binding, Figure 10B (i)), gallium-based LMs nanocarriers are distinguished by their ability to achieve flexible drug transport (*via* both intracellular and intercellular routes) and active targeting through adaptive deformability and controlled actuation driven by external stimulus (Figure 10B (iii) and Figure 10D). For example, Wang et, al reported a leukocyte membrane-coated gallium nanocarrier that is capable actively of seeking, penetrating, and internalizing into cancer cells through acoustically propelled motion (Figure 10B (iii) and Figure 10D) 104. Besides, the physical/chemical changes under external stimulus may also promote the drug release from gallium-based LMs nanocarriers, such as thermal-induced drug release, ion exchange-enabled drug release, mutual fusion-induced drug release and deformation-induced drug leakage, which represents effective strategies for controlled drug release (Figure 10E) 45, 93, 104, 128, 129, 173. For instance, after intracellular internalization into the mildly acidic endosomal/lysosomal microenvironments, gallium-based LMs nanocarriers will undergo mutual fusion due to the dissolution of the surface oxide layer, thus promoting drug release (Figure 10E (iii)) 104, 173. While the external stimulus-triggered heat accumulation (e.g., magnetocaloric effect), chemical reactions (e.g., redox reactions) and shape transformations (e.g., magnetics-induced deformation) may also synergistically contribute to controlled drug release, by either disrupting drug-carrier linkages, modifying local chemical microenvironments or restructuring carrier architectures to modulate payload release kinetics (Figure 10E (i, ii, iv)) 44, 128, 129. Notably, the external stimulus-triggered shape transformations (due to oxidation changes) could also enable effective lysosomal escape, e.g., spherical-rod transformation under light irradiation, which can mechanically disrupt endosomal membrane to facilitate the endosomal escape of payloads (Figure 10E (iv)) 105. Moreover, the heat accumulation and ROS generation under external stimulus complement the limitations of solely chemotherapy, providing synergistic and complementary effects for various therapeutic modalities (detailed in the previous section: **Gallium-based liquid metal sensitizers**).

Overall, gallium-based drug carriers provide a novel approach to achieve spatiotemporally controlled intracellular drug delivery, offering broad application potential for multimodal therapeutic regimens.

## Summary and Future Perspectives

Gallium, as a multi-targeted pharmacologically active metallic element, has shown great potential in the field of medicine, especially in antibacterial, anti-inflammatory, anticancer, osteogenesis, radio-pharmacology and molecular imaging. With the emergence of various pharmaceutical formulations, including gallium compounds, gallium complexes, gallium radioisotopes, gallium-based nanomedicines, and gallium-based liquid metals, gallium-based pharmaceuticals may witness a series of breakthrough discoveries and groundbreaking technologies in terms of novel pharmaceuticals development and comprehensive applications. This review presents the basic concepts of gallium-based pharmaceuticals, with an emphasis on a systematic description of typical physicochemical properties, pharmaceutical activities, pharmaceutical formulations and practical applications.

Herein, we provide the following key challenges and perspective outlooks derived from the current trend analysis, aiming to aid future academic endeavors and clinical translation of gallium-based pharmaceuticals.

### Biosafety requires systematic exploration

Investigations into the toxicological effects and possible hazards of gallium and related derivatives are growing exponentially, with substantial evidence confirming that gallium compounds, gallium complexes, micro/nanoparticles, and gallium-based LMs are considered to be nontoxic or slightly toxic in terms of cytotoxicity, hepatotoxicity, hemotoxicity, and histotoxicity. However, the toxicological mechanisms, metabolic routes and potential hazards of gallium and related derivatives on living organisms have not yet been fully elucidated, particularly given the scarcity of clinical trials involving human patients. A comprehensive biocompatibility assessment is a prerequisite, since biosafety is related to a complex and diverse set of influencing factors, including composition, pharmaceutical formulations, dosage, and administration environment. Considering that gallium is not an essential element for living organisms, intensive research on the *in vivo* distribution, biochemical mechanisms, bioavailability and metabolic routes of gallium and related derivatives are crucial to be implemented. In addition, future research should pay more attention to the potential hazards/adverse effects of gallium and related derivatives, along with off-target biodistribution and non-specific cellular uptake, long-term impacts of residual/non-degraded gallium components and degraded products, subsequent cellular damage (e.g., apoptosis, necrosis, phagocytosis, ferroptosis, or pyroptosis) and underlying molecular mechanisms, as well as secondary genetic modulation/mutation and immune dysregulation triggered by persistent gallium exposure. In conclusion, despite the rapid progress of relevant studies, the current research is still limited to the discovery of basic phenomena, while the investigation of the toxicological effects of gallium and related derivatives on organisms is still in its infancy.

### The knowledge gaps in the action mechanisms of pharmaceutical activities

Although considerable progress has been made in elucidating the interactions of the active pharmaceutical ingredient (Ga^3+^ ions) with biological systems, there are still many issues that need further investigation. For instance, Ga (III) as a non-functional Fe (III) mimic tends to interfere with iron metabolism/iron homeostasis, which is the most well-known pharmaceutical mechanism. However, it remains uncertain whether Ga (III) may affect other iron-dependent essential biological processes in non-target cells and induce undesirable downstream effects. In addition, as more and more pharmaceutical formulations emerge, gallium-based pharmaceuticals with diverse structures, compositions and biochemical properties will show more complex biological interactions. For example, recently emerged nano-sized pharmaceutical formulations constructed from active pharmaceutical ingredient (Ga^0^), may not only retain the pharmaceutical activities of Ga^3+^ ions, but also significantly improve the biocompatibility and targetability. During the exploration of novel gallium-based pharmaceuticals, gallium and related derivatives have also exhibited diverse targets (e.g., dysregulation of cellular redox homeostasis and modulation of immune responses) rather than simply interfering with iron metabolism/iron homeostasis, as well as more complex therapeutic mechanisms (e.g., heat accumulation, ROS generation, and galvanic replacement). Therefore, an in-depth investigation of the structure-function-mechanism relationship of gallium and related derivatives will greatly contribute to the development of more efficient gallium-based pharmaceuticals.

### The impact of pharmaceutical formulations on bioavailability and efficacy deserves special attention

At the application level, the ligand modification to enhance the bioavailability and pharmacokinetic properties of active pharmaceutical ingredient Ga^3+^ ions is of particular interest, because Ga^3+^ may virtually hydrolyze to insoluble gallium hydroxide within neutral aqueous solutions. In addition to limiting the hydrolysis of Ga^3+^ ions by chelation or coordination, optimizing pharmaceutical formulations through strategies such as chemisorption, physical adsorption, bioconjugation, polymerization, or galvanic replacement, is also an effective way to improve the bioavailability and impart additional therapeutic functionality of active pharmaceutical ingredients (Ga/Ga^3+^ ions). For example, unmodified gallium droplets synthesized *via* probe sonication method exhibited almost negligible antimicrobial activity against Gram-positive bacteria methicillin-resistant *Staphylococcus aureus* (MRSA) and the Gram-negative bacteria *Pseudomonas aeruginosa* (*P. aeruginosa*), probably due to spontaneous formation of partially passivating oxide film restricting the release of Ga^3+^ ions. In contrast, antimicrobial activity can be significantly improved *via* simple surface functionalized modifications (e.g., polymers, ligands, polysaccharides, natural compounds, metals, etc.). However, how to enhance the therapeutic/diagnostic efficacy of gallium-based pharmaceuticals with different formulations by controlling the release rate of the active pharmaceutical ingredients, enhancing the bioavailability, and improving the long-term stability in the organism remains to be further investigated.

### Seeking efficient delivery strategies remains a key challenge in improving the efficacy of gallium-based pharmaceuticals

Successful drug delivery strategies must take into account a variety of factors, including administration routes, cargo loading, drug cycling, and stimulated release strategies. Among these factors, administration routes and pharmacokinetics are key in determining drug specificity and efficacy. Currently, the clinical efficacy of gallium compounds and complexes *via* intravenous administration has been demonstrated, but the optimal timing and dosages still need to be optimized. More innovative formulations of gallium-based pharmaceuticals (e.g., nanomedicines, embolic agents, implants, etc.) provide broader choices of administration routes, such as intravenous, oral, transdermal, mucosal, and implant administrations, etc., which provide new insights for improving bioavailability, realizing targeted drug delivery, and reducing side-effects. In addition, some advanced drug delivery systems (e.g., devices and appliances), such as microneedle patches, needleless injection devices, micropumps, etc., also show promising compatibility with gallium-based pharmaceuticals and deserve more attention. However, these novel delivery systems also face challenges in terms of stability, safety and functionalization, which require further research and improvement. Therefore, future studies should focus on exploring the optimal administration routes, timing and dosages for the administration of different gallium-based pharmaceuticals, and comprehensively evaluating their pharmacokinetic properties, in order to seek more efficient drug delivery strategies.

### Breaking up the barriers in translational medicine of gallium-based pharmaceuticals

In order to comprehensively explore the inherent opportunities, potentialities and challenges for translational gallium-based pharmaceuticals, it is necessary to pay close attention to four pivotal phases: innovation, implementation, preclinical trials and early/late clinical trials 28. Currently, in the bench phase, the distinctive properties of liquid metal gallium have empowered researchers to develop a myriad of gallium-based pharmaceuticals, bioactive materials, drug delivery systems, and medical assistive devices with significant pharmaceutical activities and exclusive benefits. With increasing attention, innovative concepts and laboratory achievements are laying the groundwork for clinical adaptation. During the 'bench to bedside' process of gallium-based pharmaceuticals, a holistic approach should be taken beyond the focus on laboratory studies, harmonising clinical needs with ongoing research to highlight advances and challenges in real-world clinical settings. Despite the confidence inspired by marketed gallium-based pharmaceuticals (mainly with Ga(III) as active pharmaceutical ingredient), there are still series of barriers that need to be confronted and overcome in the clinical translation of newly developed liquid metal gallium-based pharmaceuticals (metallic Ga(0) as active pharmaceutical ingredient). Therefore, it is crucial to strengthen scientific research, technological innovation, comprehensive policymaking, and collaborative advocacy to facilitate widespread clinical translation of gallium-based pharmaceuticals.

### Future outlooks

Despite the existing challenges that remain to be addressed, the sustained and rapid advancements in this field herald a bright future for gallium-based pharmaceuticals. Future research should prioritize the following strategic directions, such as systematic biosafety assessments tailored to specific formulations and administration routes, in-depth exploration of complex mechanisms (e.g., interactions with molecules, targets, cells, organs and organisms) to elucidate pharmaceutical activities, nanotechnology-enabled innovation in pharmaceutical formulations engineering, optimization of drug delivery regimens to revolutionize therapeutic strategies, multidisciplinary collaboration to refine research frameworks (e.g., technological and theoretical systems) and overcome clinical translational obstacles (e.g., regulatory and policy constraints, clinical application scenarios, large-scale preparation technologies, production costs and sustainability).

Ultimately, we believe this timely review will effectively inspire interdisciplinary collaborations and innovations in the field of gallium-based pharmaceuticals, which may promote systematic and comprehensive research in the future, thereby advancing the sustainable development of these cutting-edge metallic pharmaceuticals.

## Figures and Tables

**Table 1:** Summary of various gallium-based pharmaceuticals.

Classification	Types		Chemicals/Polymer	Other Substance added/formed (and their Purpose/Role)	Administration routes	Particle size	Properties	Specific application types	*In Vivo* dosage	Year	Ref.
Therapeutic agents	Gallium (III) compounds		Gallium nitrate	None	None (*In Vitro*)	None	Bone regeneration	Osteoblast and Osteoclast cells	0.1-100 μg/mL	1990	13
					Intravenous administration	None	Antibacterial	NHL	200-300mg/m^2^/d	2004	87
					Intravenous administration	None	Antibacterial	*P. aeruginosa*	IC_90_=10μM	2007	88
			Gallium chloride	None	Intravenous administration	None	Antitumor	Lung cancer	IC_50_=100 μM ~ 1 mM	1989	89
	Gallium (III) complexes		Gallium maltolate	None	Intravenous administration	None	Antitumor	HCC	IC_50_=10 ~ 40 μM	2006	90
			KP46	None	Oral administration (*In vivo* preclinical studies)	None	Antitumor	Breast cancer	IC_50_=1 ~ 3 μM	2009	91
	Gallium-based nanomedicine		ICG-Ga NPs	None	Intravenous administration	5~15 nm	Antibacterial; Anti-inflammatory	*ESBL E. coli* (Pyogenic liver abscess and keratitis)	25 μg/mL	2021	23
			GaTa-CP NPs	Tannin (assits Ga^3+^ in antibacterial action)	Inhalation	~250 nm	Antibacterial; Antitumor	*E. coli*, *S. aureus*, *S.Intermedius*, and *P. intermedia* in Lung cancer	Ga^3+^ :10 μg/mL	2023	25
			LMNDs	DOX (stabilization)	Multisite intra-tumoral administration	~20 nm	Antitumor	Breast cancer (BCap-37) cells	IC_50_= 30.4 µg/mL	2023	26
			LM-EGCG	mPEG-SH (stabilization)	Oral administration	~250 nm	Antibacterial; Anti-inflammatory	*Escherichia_Shigella* (Inflammatory bowel disease)	Ga= 5mg/kg	2024	27
			DPMG	DSPE-PEG2000-Mal (enhance antigen adsorption)	Intravenous administration	~130 nm	Antitumor	4T1 cells	1 mg/mL, 50 μL	2023	43
	Gallium-based LMs sensitizers	Photo	LM@GOX	mPEG-SH (stabilization); GOX: (catalysis)	Intravenous administration	98.21nm	Antitumor	4T1 cells	300 μg LM and 30 μg GOX	2019	92
			LM@MSN/DOX@HA	HA (targeting); DOX (antitumor); MSN (delivery carrier)	Intravenous administration	160.4nm	Antitumor	4T1 cells	Ga concentration at 150μg/mL, 100μL	2019	93
		Magnetic	GLM-Fe	Fe (magnetic iron particle)	None (*In Vitro*)	∼200 nm to ∼2 μm	Antibacterial	*P. aeruginosa*, *S. aureus*	100 μg/mL	2020	56
		Microwave	PEG-IL-LM-ZrO_2_ SNPs	PEG (stabilization); IL (MW sensitive effect)	Tail vein injection	210 ± 60 nm	Antitumor; CT imaging	H22 tumor cells; Hepa 1-6 tumor cells	Maximum dose= 20 mg/mL (50, 100, 150 mg kg^-1^ *In Vivo*)	2019	94
		Thermal	Ga/M/PPs	C8161 cell membrane (targeting); Paclitaxel (antitumor)	Intratumoral injection	1 μm	Antitumor; CT imaging	C8161 cells	500 mg/kg	2022	95
		Chemical	LM-Pd	LM (modulating biorthogonal catalysis); Pd (catalyst)	Intravenous administration	~ 210 nm	Antitumor	CT26 cells	16 mg/kg, 200 μL	2023	96
Therapeutic agents	Gallium-containing bioactive materials	Bone regeneration scaffolds	Gallium-doped hydroxyapatite	(NH_4_)_2_HPO_4_ (preparing sample)	None (only the material preparation part is involved)	2 μm	Osteosynthesis and calcium retention in loco.	None	11.0 mass% Gallium ions	2009	97
		Dental/Orthopaedic implants	EGaIn	None	None (*In Vitro*)	None	Antibacterial	*E. coli*, *S. aureus*	Ga: 0.377 ± 0.015, 0.390 ± 0.026, and 0.483 ± 0.025 μmol/mL for 4, 8, and 24 h	2020	54
			Ga-Ti-Al-Zr-Si	None	None (*In Vitro*)	None	Antibacterial	*MRSA*	Ga: 1, 2, 20, and 23 *wt*%	2019	98
		Hemostatic composites	Ga-MBG/CHT scaffolds	Chitosan (hemostat)	None (*In Vitro*)	Average pore diameter: 6~12 nm	Hemostatic function; Antibacterial	Human blood; *E. coli*, *S. aureus*	molarratio: Si/Ca/P/ Ga=79:15:5:1	2017	99
Diagnostic agents	Gallium radioisotopes		^68^Ga-FAPI-2 and ^68^Ga-FAPI-4	FAPI-2/FAPI-4 (targeting)	Intravenous injection	None	CT and PET imaging; Antitumor	Breast cancer, Colorectal cancer,etc.	The effective dose of ^68^Ga-FAPI-2 and ^68^Ga-FAPI-4 PET (1.4-1.8 mSv/100 MBq)	2019	22
	New generation of Gallium-based diagnostic agents		Ga	None	Interventional local drug administration	~100 µm	X-ray images	Pig hearts and kidneys	0.8-1mL	2014	100
			Fe@EGaIn/CA	CA (embolization and drug-loading); DOX · HCl (antitumor)	Interventional local drug administration	~500 µm	CT imaging and MRI; Antitumor	VX2 cancer cells	100 μL saline suspension containing about 50 μL microspheres	2021	44
			GMs	Chitosan	Intratumor injection	~200 µm	CT imaging and MRI; Antitumor	C8161 tumor cells	200μL GMs-CS mixture (40μL Gallium)	2020	101
			GaNP/Si	Si wafers (depositing Ga)	None (*In Vitro*)	< 50 nm	DNA sensing	Peripheral blood leukocytes from cystic fibrosis patients	None	2016	102
			DSPE-PEG2000-Amine-DC(8,9)PC-LM	DSPE-PEG2000-Amine (Surfactant); DC(8,9)PC (Surfactant)	Intratumor injection	∼150 nm	PA imaging; Antitumor	EGFR-positive human colon adenocarcinoma HT29 cells	10 mg/mL	2017	33
Gallium-based drug carrier	Gallium (III)-based drug carrier		ADR-Ga-Tf	Tf; DOX (Antitumor)	None (*In Vitro*)	None	Antitumor	MCF-7/ADR cells	IC_50_= 9.52 x 10^-2^ μM	2000	103
	Gallium-based LM drug carrier		LMGNS	Leukocyte Membrane (active targeting); DOX·HCl (Antitumor)	None (*In Vitro*)	~7 μm in length; diameters of ~800 nm and ~150 nm at each end (needle-like shape)	Antitumor	HeLa cells	10 μg/mL	2020	104
			tNPs	Graphene quantum dots (control particle size; absorb photoenergy and generate local heat and ROS) and DOX (antitumor)	Intravenous injection	~100 nm	Antitumor	Cervical cancer tumor	Dox/tNP: IC_50_ = 0.35 mg/L (Dox concentration)	2017	105

Note: NHL, *Non-Hodgkin's Lymphoma*; *P. aeruginosa, Pseudomonas aeruginosa*; HCC, *Hepatocellular carcinoma*; Gallium maltolate, *Tris(3-hydroxy-2-methyl-4H-pyran-4-onato)Gallium*; KP46,*Tris (8-quinolinolato) gallium)*; ICG, *Indocyanine Green*; *ESBL E. coli, Extended Spectrum Beta Lactamase Escherichia coli*; CP, *capsular polysaccharide*; Ta, *Tannin*; *E. coli*, *Escherichia coli*; *S. aureus*, *Staphylococcus aureus*; *S. Intermedius*,* Streptococcus. Intermedius*; *P. intermedia*,* Prevotella intermedia;* LMNDs, *liquid metal nanodroplets*; DOX, *Doxorubicin*; LM, *liquid metal*; EGCG, *Epigallocatechin gallate*; DSPE-PEG2000-Mal, *1,2-Distearoyl-snglycero-3-phosphoethanolamine-N-[maleimide(polyethylene glycol)-2000]*; PEG, *Polyethylene Glycol*; GOX, *glucose oxidase*; MSN, *mesoporous silica nanoparticle*; HA, *Hyaluronic acid*; GLM, *Galinstan-based liquid-metal*; IL, *Ionic liquid*; SNPs, *supernanoparticles*; MW, *microwave*; Ga/M/PPs, *Membrane coated Ga particles with paclitaxel*; MRSA*, multidrug resistant Staphylococcus aureus*; MBG, *mesoporous bioactive glass*; CHT, *chitosan composite scaffolds*; FAPI, *Fibroblast activation protein inhibitors*; CT, *computed tomography*; PET, *positron emission tomography*; CA, *calcium alginate*; MRI, *magnetic resonance imaging*; GMs, *Gallium microparticles*; CS, *Chitosan*; DSPE-PEG2000-Amine, *1,2-distearoyl-sn-glycero-3-phosphoethanolamine-N-[amino(polyethylene glycol)-2,000]*; DC(8,9)PC, *1,2-bis(10,12-tricosadiynoyl)-sn-glycero-3-phosphocholine*; PA, *photoacoustic*; EGFR, *epidermal growth factor receptor*; MDR, *multidrug resistance*; Tf, *Tfansferrin*; LMGNs, *leukocyte membrane-coated gallium nanoswimmer*; tNPs, *transformable liquid-metal nanoparticles*; GQDs, *graphene quantum dots*; ROS, *reactive oxygen species*.
